# *Corydalis Saxicola* Bunting Total Alkaloids Attenuate Walker 256-Induced Bone Pain and Osteoclastogenesis by Suppressing RANKL-Induced NF-κB and c-Fos/NFATc1 Pathways in Rats

**DOI:** 10.3389/fphar.2020.609119

**Published:** 2021-01-26

**Authors:** Linjie Ju, Peipei Hu, Ping Chen, Jiejie Wu, Zhuoqun Li, Zhixia Qiu, Jun Cheng, Fang Huang

**Affiliations:** ^1^ Department of Chinese Pharmacology and Traditional Chinese Medicine, China Pharmaceutical University, Nanjing, China; ^2^ Nanjing Zhongshan Pharmaceutical Co, Ltd., Nanjing Economic and Technological Development Zone, Nanjing, China

**Keywords:** *Corydalis saxicola* bunting total alkaloids, cancer induced bone pain, osteoclastogenesis, IκBα, RANKL induced NF-κB and c-fos/NFATc1 pathways

## Abstract

Metastatic bone pain is characterized by insufferable bone pain and abnormal bone structure. A major goal of bone cancer treatment is to ameliorate osteolytic lesion induced by tumor cells. *Corydalis saxicola* Bunting total alkaloids (CSBTA), the alkaloid compounds extracted from the root of *C. saxicola* Bunting, have been shown to possess anticancer and analgesic properties*.* In this study, we aimed to verify whether CSBTA could relieve cancer induced bone pain and inhibit osteoclastogenesis. The *in vivo* results showed that CSBTA ameliorated Walker 256 induced bone pain and osteoporosis in rats. Histopathological changes also supported that CSBTA inhibited Walker 256 cell-mediated osteolysis. Further *in vitro* analysis confirmed that CSBTA reduced the expression of RANKL and downregulate the level of RANKL/OPG ratio in breast cancer cells. Moreover, CSBTA could inhibit osteoclastogenesis by suppressing RANKL-induced NF-κB and c-Fos/NFATc1 pathways. Collectively, this study demonstrated that CSBTA could attenuate cancer induced bone pain via a novel mechanism. Therefore, CSBTA might be a promising candidate drug for metastatic bone pain patients.

## Highlights

*Corydalis saxicola* Bunting total alkaloids ameliorate Walker 256-induced bone pain and osteoclastogenesis in rats.
*Corydalis saxicola* Bunting total alkaloids inhibit osteoclastogenesis via suppressing RANKL induced NF-κB and c-Fos/NFATc1 pathways.
Inhibiting the elevated formation or activity of osteoclasts is a valuable strategy against cancer induced pathological osteolysis and bone pain.

## Introduction

Metastatic bone pain is one of the most common forms of cancer induced unrelenting pain which significantly reduces patients’ living quality (Mao-Ying et al., 2006; Dai et al., 2017). Although with good hospice palliative care, patients with bone metastasis in later stage will suffer from excruciating pain due to the peripheral and neuropathological mechanisms of cancer induced bone pain. Strangely, primary breast tumors at the original site led to less or no cancer pain, while excruciating and persistent pain made patients suffer from both physical and psychological ‘hits’ once cancer cells metastasized to bone (Falk and Dickenson, 2014). Meanwhile, the precise mechanisms about how breast and other cancer cells induce pain remain a challenge for the researchers (An et al., 2018).

Osteoclastic activity is related to CIBP, and cancer invasion can induce significant proliferation of osteoclasts (Park et al., 2018). Physiologically, osteoclasts utilize acidity to solubilize the mineralized fraction of the bone matrix with tight control to avoid protons leaking and ineffective bone resorption. Once tumor cells (e.g., breast, thyroid, lung, and renal cancers) metastasized to the bone, the aforementioned cancer-induced osteoclastogenesis leads to the excessive acidity in the bone marrow by increasing absolute osteoclast number. Simultaneously, bone is a richly innervated tissue, and the acidity overload induced ‘bays’ and ‘pits’ between the osteoclasts and bone started to stimulate the transient receptor potential vanilloid subfamily, member 1 (TRPV1) in the sensory nerve fibers of bone, ultimately inducing CIBP (Yoneda et al., 2011; Fornetti et al., 2018). However, tumor cells themselves do not destroy bone structure but rather secrete excessive receptor activator of nuclear factor κ-B ligand (RANKL). RANKL binds to its natural receptor RANK expressed on the preosteoclasts so as to promote osteoclast maturation and finally result in exacerbated bone resorption (Luo et al., 2016). Collectively, cancer cells promote bone pain via an indirect osteoclastic acid-induced stimulation of nociceptors in bone as well as osteolytic lesion that, in turn, foster hyperalgesia through the release of inflammatory factors (Aielli et al., 2019). The process whereby metastatic cancer cells induced osteolytic lesions to aggravate osteoclastic activity and bone pain is more like a ‘vicious cycle’. Osteoprotegerin (OPG), a soluble decoy RANKL receptor secreted by osteoblasts, can sequester RANKL and inhibit the recruitment as well as the formation of osteoclasts. The balance between RANKL and OPG usually regulates the normal structure of bone (Lacey et al., 2012). Studies also suggested that administration of the OPG to osteosarcoma-bearing animals significantly decreased spontaneous pain behaviors without affecting the tumor size (Honore et al., 2000; Luger et al., 2001). To put it in a nutshell, osteoclast plays a pivotal role in CIBP.

In the past decades, radiotherapy is the cornerstone treatment for CIBP in the clinical setting; opioids can ameliorate CIBP, however, high dosages and/or long-term usage always result in seriously adverse effects; radiopharmaceuticals, such as strontium and samarium, are effective but myelosuppressive; and non-steroidal anti-inflammatory drugs (NSAIDs) often present insufficient effect. Previous CIBP studies commonly regarded the ion channels in the bone sensory nerve as the regulatory factors, but researches about inhibiting osteoclasts to alleviate CIBP have been paid more attention in recent years (Fornetti et al., 2018; Aielli et al., 2019). Inhibition of the osteoclast formation is a promising strategy for CIBP treatment. For example, bisphosphonates are widely used to relieve bone destruction and augment analgesic efficacy by reducing the osteoclast activity or promoting osteoclast apoptosis in patients with breast cancer (Goldvaser and Amir, 2019).

Compared with the core drugs of WHO “Three Step Therapy”, traditional Chinese medicine or natural products may be preferred and effective therapeutic choices for CIBP with fewer side-effects (Cong et al., 2015). *Corydalis saxicola* Bunting total alkaloids (CSBTA), the alkaloid compounds extracted from the root of *C. saxicola* Bunting, have been shown to possess anticancer and analgesic properties (Zhang et al., 2016; Yu et al., 2018). The active ingredients of CSBTA mainly consist of dehydrocarbamate, palmatine and berberine (Yu et al., 2018; Kuai et al., 2020). Some recent reports have shown that CSBTA could alleviate hepatitis, tumors and neuropathy by its anti-inflammation effect (Zeng et al., 2013; Zhang et al., 2016; Wu et al., 2017; Fu et al., 2018). Pain is one of the cardinal features of inflammation. CIBP caused by breast cancer, generally, was closely related to preosteoclasts inflammation *in vivo* (Park et al., 2018). However, researches on the alleviation of CIBP by CSBTA has been rather lacking. In this study, our team aimed at verifying whether CSBTA could attenuate CIBP by restraining osteoclast activation *in vivo* and *in vitro*.

## Materials and Methods

### Drugs, Chemicals and Reagents

TritonX-100, Dimethyl sulfoxide (DMSO) and Tween-20 were purchased from Sigma–Aldrich (Sigma-Aldrich, USA). N-acetyl-leu-leu-norleucinal (ALLN) with a purity>98% was obtained from APExBIO Co., Ltd. (Houston, USA). Soluble receptor activator of nuclear factor-κB Ligand (sRANKL) and macrophage colony stimulating factor (M-CSF) were purchased from Novoprotein biotechnology (Shanghai, China). RIPA lysis buffer, QuickBlock™ blocking buffer, SDS-PAGE sample loading buffer (6X), antifade mounting medium with DAPI, Alexa Fluor 488-labeled goat anti-rabbit IgG (H + L), horseradish peroxidase (HRP)-labeled goat anti-rabbit or goat anti-mouse IgG conjugates, MTT cell proliferation and cytotoxicity assay kit and RIPA lysis buffer were from Beyotime Co. (Shanghai, China). The primary antibodies against RANKL (ab45039), Osteoprotegerin (OPG, ab9986) and TRPV1 (VR1, ab10296) were purchased from abcam (United Kingdom). Antibodies against IκB-α (sc-371), p-IκB-α (sc-8404) and NF-κB p50 (sc-8414) were from Santa Cruz biotechnology (CA, USA). Antibodies against NF-κB p65 (#8242), Phospho-NF-κB p65 (Ser536) (#3033), NFATc1 (#8032) and c-Fos (#2250) were from Cell Signaling Technology (Danvers, MA, USA). Antibody against Tubulin-β (AP0064) were from Biogot technology, co, Ltd. (Nanjing, China). PCNA Polyclonal Antibody (10205-2-AP) were from Proteintech Group, Inc. (Wuhan, China). Medium 199 (M199), Dulbecco’s modified Eagle’s medium (DMEM), F-12 medium, Roswell Park Memorial Institute (RPMI)-1,640 medium, fetal bovine serum (FBS), horse serum, penicillin-streptomycin solution (100X), bovine serum albumin (BSA), 0.25% trypsin-ethylenediaminetetraacetic acid (trypsin–EDTA) were purchased from Gibco Life Technologies Corp. (USA). Total RNA extraction reagent (Trizol) was obtained from Vazyme Biotech (Nanjing, China). Zoledronic acid injection was purchased from Chia tai tianqing pharmaceutical group Co., Ltd. (0.8 mg/ml, Lianyungang, China).

### Preparation and Quality Control of *Corydalis saxicola* Bunting Total Alkaloids

*Corydalis saxicola* Bunting total alkaloids (CSBTA) were provided by Nanjing Zhongshan Pharmaceutical Co., Ltd. (Batch number: 191201). In brief, the quantitative analysis of CSBTA by high-performance-liquid chromatography (HPLC) were performed by SHIMADZU LC-20 AT HPLC system (SHIMADZU, Japan) equipped with a quaternary ammonium salt pump solvent management system, an on-line degasser and an autosampler. The separation was conducted by an Agilent ZARBAX SB-C18 column (USA, 4.6 × 250 mm, 5 μm) with the 10 μL injection volume and the column temperature was maintained at 30°C. The mobile phase was composed of acetonitrile (A) and potassium dihydrogen phosphate solution (B, 0.01 mol/L), and gradient elution was set as 25% A: 75% B with the 1 ml/min flow rate. The detection was performed as 347 nm. Quantitative analysis was obtained by external standard method. There were about 27 peaks in HPLC profile, and the major three peaks were dehydrocarbamate, palmatine hydrochloride and berberine hydrochloride with the content of 13.79, 8.38, 1.52%, respectively (Supplementary Figure S1). For the *in vitro* experiments, 0.2 g CSBTA powder was dissolved in 4 ml DMSO as the stock solution.

### Animals

Healthy female Wistar Han (HsdBrlHan) rats were provided by Shanghai SIPPR/BK Experimental Animal Co., Ltd. (SCXK 2013-0016). The rats were housed with controlled temperature 23 ± 2°C, 45–75% relative humidity, and 12 h light-dark cycle. All the animal experiments were conducted between 08:00 a.m. and 16:00 p.m. according to the International Association for the Study of Pain (IASP) and Provisions and General Recommendation of Chinese Experimental Animals Administration Legislation. Approval of experimental procedures was accepted by the Animal Ethics Committee of China Pharmaceutical University.

### Preparation of Carcinoma Ascites Fluid Cells

Walker 256 rat mammary gland carcinoma cells were purchased from the Cobioer company (Nanjing, China) and cultured with Medium 199 (supplemented with 5% horse serum) at CO_2_ incubator (5% CO_2_: 95% filtered air, ThermoFisher). Then 0.5 ml Walker 256 cell suspension (10^7^ cells/ml) was injected into the abdominal cavity of female Wistar rats (60–80 g). The ascitic fluid was harvested by abdominal puncture from the rats after 1 week, centrifuged and washed by Hank's solution (KeyGen, Nanjing, China) for three times to get the cells. Finally, the sediment was resuspended to a density of 1×10^5^ cells/ml with Hank's solution at 4°C until model establishment of CIBP.

### Establishment of Cancer Induced Bone Pain Model

Female rats (160–180 g) were acclimatized for a week with free access to food and sterile water, then they were randomly divided into five groups: Sham group (equal normal saline solution, i. g.), CIBP group (Cancer induced bone pain group, equal normal saline solution, i. g.), CSBTA (L) group (L, 25 mg/kg/day, i. g.), CSBTA (M) group (M, 50 mg/kg/day, i. g.), CSBTA (H) group (H, 100 mg/kg/day, i. g.), Zoledronic acid (Zol, 250 µg/kg/day, intravenous injection) (Nagae et al., 2007). Briefly, rats were anesthetized by sodium pentobarbital (i.p. 50 mg/kg). Then superficial incisions were made in the skin overlying the anterior border of right tibiae. Dental drill (Φ 0.8 mm) was inserted at the site of mid-shaft region of the tibia and pierced into the medullary cavity. Tumor cell suspension (15 μL) was slowly injected into cavity of the tibia by the micro-syringe (Hamilton, USA). Then dental sealants were used to avoid the leak of tumor cells from the wound immediately. For the Sham group, 15 μL Hank’s solution was injected into the medullary of the rat tibiae as control. The incision was stitched and sprinkled with penicillin powder and all the rats were allowed to recover from the anesthesia state. Incidentally, the hindpaw of the injected side was defined as the ‘ipsilateral’ hindpaw, and the non-injected side was termed as the ‘contralateral’ hindpaw.

### Limb Use Test

The rats were allowed to move freely around in a transparent standard cage without bedding (200 × 50 × 50 cm) at room temperature (Falk et al., 2019). After 10 min acclimation, each animal was observed for 3 min and the limb use score from four to zero was assigned based on the use of the ipsilateral hind limb as Supplementary Table S1.

### Von Frey Test

Before the test, rats were placed independently in small stainless-steel cages with penetrable bottom and habituated to the housing facilities for 15–30 min. Then a monofilament was applied perpendicularly to the plantar surface of the rat hind paw until it buckled like the shape of C. It was considered as the positive response if the rats exhibited any nociceptive reflexes such as paw licking, shaking, rapid paw withdrawal during the test or soon after the test. The result is quantified according to the Dixon’s “up-and-down” method (Dixon, 1980). If there was no response after 3 s of stimulation, the next filament with a higher force than before would be applied. On the contrary, a paw withdrawal response observed within the limits of 3 s represented the application of a lower force filament. Investigators should repeat above procedures until six readings were obtained after the first positive reaction in blind method, and the outcomes ("O" for no response or "X" for response) were also recorded. Tests were repeated for three times in one animal with 5 min interval at least, and the average figures were used as the final data.

### Histological Examination and Identification

Rats were anesthetized with overdose of sodium pentobarbital. Then rat tibiae and lumbar 4 (L4) -L5 dorsal root ganglions (DRGs) were fixed by immersing in 4% paraformaldehyde solution (Biosharp, Beijing, China). After decalcification, tibiae and DRGs were dehydrated and embedded in paraffin for cutting into 8 µm sections via the rotary microtome (Carl Zeiss, Germany).

For tibia hematoxylin-eosin staining and tartrate-resistant acid phosphatase (TRAP) staining, the sections were stained with HE staining kit (Beyotime, Shanghai, China) and Acid phosphatase, Leukocyte (TRAP) kit (387A, Sigma-Aldrich, Germany) following the manufacturer's protocol.

For the immunohistochemical staining, tibia and DRG sections were incubated with methanol containing 3% H_2_O_2_ for 15 min. After antigen repair, sections were blocked with 10% goat serum (Gibco) in TBS for 3 h. Then sections were incubated with primary antibody at 4°C overnight. After washing and horseradish peroxidase enzyme labeled secondary antibodies incubation, DAB substrate solution and hematoxylin was used according to the previous report (Hao et al., 2018).

### Cell Lines

Osteoclast precursor RAW 264.7 cells and human breast cancer cell lines (MDA-MB-231) were purchased from American Type Culture Collection. RAW 264.7 cells were cultured in Dulbecco's modified essential medium supplemented with 10% FBS and 1% penicillin-Streptomycin Solution. MDA-MB-231 cells were cultured in Leibovitz’s L-15 medium supplemented with 10% FBS and 1% penicillin-Streptomycin Solution.

### Osteoclast Differentiation Assay

The effect of CSBTA on osteoclast differentiation was assessed via TRAP staining and with three different protocols as follows: (i) addition of conditioned media (CM) from Walker 256 to RAW 264.7 cells for 5 days (ii) addition of CM from MDA-MB-231 to RAW 264.7 cells for 5 days (iii) addition of sRANKL (100 ng/ml) and M-CSF (50 ng/ml) to RAW 264.7 cells for 5 days.

For the osteoclast differentiation induced by Walker 256 cell CM, Walker 256 cells were cultured by serum-free RPMI 1640 medium at the density of 10^4^ cells/mL for another 48 h. Then the CM was obtained and stored at −80°C. For the (ii) protocol about stimulation of osteoclast differentiation by MDA-MB-231CM. MDA-MB-231 CM was harvested as the (i) protocol. After CM collection, RAW 264.7 cells were plated in 24-well plates at a density of 3,000 cells per well and subsequently replaced by α-MEM medium contained with 50% CM. TRAP staining was performed after the stimulation of CM to evaluate the effect of CSBTA on osteoclastogenesis.

### Cell Viability Assay

The CSBTA concentration of cell experiment was confirmed by the MTT kit. RAW 264.7, MDA-MB-231 and Walker 256 cells were planted in 96-well plates, pre-incubated with 100 μL culture medium containing different concentrations of CSBTA and DMSO, then placed in the incubator for another 24 h. Then each well was added with 10 μL MTT solution (5 mg/ml) for another 4 h in the incubator. Finally, the culture medium was replaced by 150 μL DMSO per-well. The absorption at 570 nm was tested by multi-function microporous plate reader (Thermofisher, USA).

### Western Blot

MDA-MB-231 and Walker 256 cells were treated with CSBTA (12.5, 25, 50 μg/ml respectively) for 6 or 12 h (for detection of RANKL and OPG). RAW264.7 cells were pretreated with CSBTA for 30 min, then stimulated with sRANKL (10 nM) with or without N-acetyl-leu-leu-norleucinal (ALLN, 50 μg/ml) for 12 h (for detection of p-IκB-α, p-NF-κB p65, c-Fos and NFATc1). Whole cell lysates and DRG tissue homogenate were obtained by RIPA lysis solution. Nuclear and cytosolic fractions were extracted by Nuclear and Cytoplasmic Protein Extraction Kit (Wanleibio, Shenyang). Whereafter, the protein concentration was measured by Enhanced BCA Protein Assay Kit (Beyotime, Shanghai). After SDS-PAGE electrophoresis, electro-transfer to PVDF membrane and membrane blocking, the PVDF blotting membranes were incubated with primary antibodies overnight at 4°C. After washed by TBST, all the blotting membranes were incubated with horseradish-peroxidase-linked IgG peroxidase. Gray-scale value of bands was visualized and analyzed by Tanon ChemImaging Systems (Tanon Tech. Co., Shanghai, China).

### Immunofluorescence

Firstly, RAW264.7 cells were plated into 12-well plates pre-embedded with coverslip for 12 h. Then the cells were pretreated with different concentrations of CSBTA (12.5, 25, 50 μg/ml) followed by stimulation of sRANKL (100 ng/ml) for another 12 h. Soon afterward the cell slides were immersed with 4% paraformaldehyde to fix for 15 min at room temperature. Washed the slides, blocked in blocking buffer and then the slides were incubated by the diluted primary antibody overnight at 4°C. Incubated the slides in fluorochrome-conjugated secondary antibody for 90 min at room temperature in the dark room. Covered the slides with antifade mounting medium with DAPI. Fluorescence was captured through the MEA53200 microscope (Nikon, Japan) equipped with a digital camera.

### Enzyme-Linked Immunosorbent Assay

Tibia homogenates were prepared as previous study (Hao et al., 2018). After quantitated by BCA protein assay kit (Beyotime, Shanghai), the level of RANKL and OPG in the tibia homogenates were measured by Enzyme-Linked Immunosorbent Assay kit (ELISA, MultiSciences, Hangzhou, China). The level of RANKL and OPG in rat serum, Walker 256 and MDA-MB-231 cell culture media were determined by ELISA kit according to the manufacturer’s protocol (Elabscience, Wuhan, China). Absorbance values were obtained by microplate reader (Thermo Fisher Scientific, USA) and the RANKL and OPG concentration were calculated from the corresponding standard curves.

### RNA Extraction and Real-Time Polymerase Chain Reaction (PCR)

After treatment by CSBTA (12.5, 25, 50 μg/ml) or DMSO for 6/12 h, cells were washed by cold PBS. The total RNA was extracted by Trizol soultion. First-strand cDNA chain was synthesized from 1 mg of total RNA by the 5X All-In-One RT Master Mix (abm, Canada). PCR primers were designed by Sangon Biotech (Shanghai, China). The endogenous controls in these assays were glyceraldehyde 3-phosphate dehydrogenase (GAPDH). Polymerase chain reaction was performed via Hieff UNICON^®^ Power qPCR SYBR Green Master Mix (Yeasen, Shanghai, China) at 95°C (15 s), 60°C (60 s) for annealing and 72°C (30 s) for 35 cycles. Relative gene expression to the Vehicle group was estimated by the ΔΔCt method. All the primer sequences were listed in Supplementary Table S2.

### Data Analysis

Data was presented as means ± standard deviation (SD). All the assays were performed in triple independent experiments at least. Statistical evaluation was performed by Student’t *t*-test or one-way analysis of variance (ANOVA, for multiple group comparisons), followed by least significant difference (LSD) post hoc test (SPSS 22.0 for Windows, SPSS Inc., USA). *p* values *<* 0.05 were considered as statistically significant.

## Results

### Effect of CSBTA Treatment on the Body Weight, Limb Usage and Mechanical Allodynia in Rats

**FIGURE 1:**
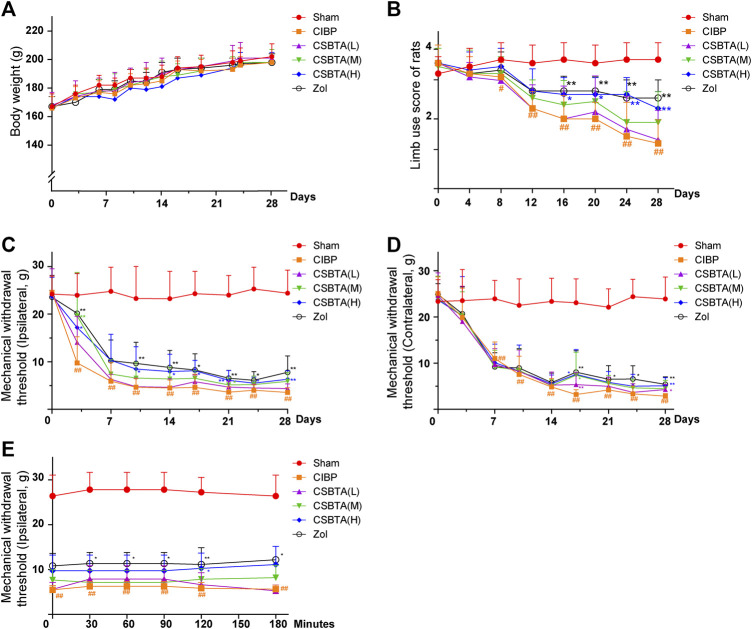
Effect of CSBTA treatment on the body weight, limb usage and mechanical allodynia in rats. Rats were dosed with CSBTA (25, 50, 100 mg/kg/day) and Zol (250 µg/kg/day, intravenous injection). **(A)** The body weight of rats (*n* = 7). **(B)** The limb use score during spontaneous ambulation (*n* = 7). **(C)** Mechanical withdrawal threshold changes of the rat ipsilateral hind paws in different groups (*n* = 7). **(D)** Mechanical withdrawal threshold changes of the rat contralateral hind paws in different groups (*n* = 7). **(E)** On the 14th day, Von-Frey test was performed at 0, 0.5, 1.0, 1.5, 2.0, 3.0 h after a single administration of CSBTA (*n* = 5). Data was presented as means ± SD. #*p* < 0.05, ##*p* < 0.01 *vs.* the Sham group. *p < 0.05, **p < 0.01 vs. the CIBP group.

As our team reported before (Kuai et al., 2020), CSBTA has strong analgesic effect on cisplatin-induced neuropathic pain rats, here the dose of 100, 50, and 25 mg/kg CSBTA by oral gavage daily were chosen in our study. As shown in Figure 1A, CSBTA could not significantly change the ascending trend of body weight during the experiments. No detectable changes were observed in the body weights of CSBTA treated or CIBP rats compared with the Sham group. Moreover, mental status of CSBTA treated rats were better, and their fur was thicker and smoother, which partly reflected the fact that administration of CSBTA exerted no apparent toxicity for the rats. Compared with the Sham group, impaired limb usage was observed in the CIBP rats with significant limping (indicated by the red line), slow-moving behaviors and partial nonuse of the ipsilateral hindlimb on the 13th day (Figure 1B). However, the symptoms of lameness mentioned above were improved in the group CSBTA (H) after the 16th day (Figure 1B, blue line). These findings indicated that CSBTA was able to ameliorate CIBP without obvious side effects.

Since the ninth day was considered as the representative day of CIBP (An et al., 2018), here we performed Von Frey test to investigate the mechanical withdrawal threshold (MWT) of rats. Apparently, the MWTs of ipsilateral hind paw in CIBP rats showed dramatic decreases from the third day to the 30th day compared with the Sham group (Figure 1C, yellow line). In contrast, after 9 days of treatment, CSBTA (H) (blue line) and CSBTA(M) (green line) significantly ameliorated tumor-induced allodynia (Figure 1C). Tissue or nerve injury in one side of the body leads to pain in the mirror-image site, namely mirror-image pain, which often occures in CIBP (Lee et al., 2005; Bloom et al., 2011). As shown in Figure 1D, CIBP significantly reduced the MWTs of the contralateral hindpaws of rats on the seventh day compared with the Sham group (Figure 1D, yellow line). While the mirror-image pain was slightly relieved after CSBTA treatment since the 15th day. However, it was obtained that the analgesic effects of CSBTA in ipsilateral hind paws shown no significant peak at 0, 0.5, 1.0, 1.5, 2.0, 3.0 h after a single administration of CSBTA on the 14th day (Figure 1E). Therefore, we postulated that CBSTA was not a short-term painkiller, it might relieve CIBP through a particular way.

### Histopathological Changes in the Tibiae and DRGs of CIBP Rats

**FIGURE 2:**
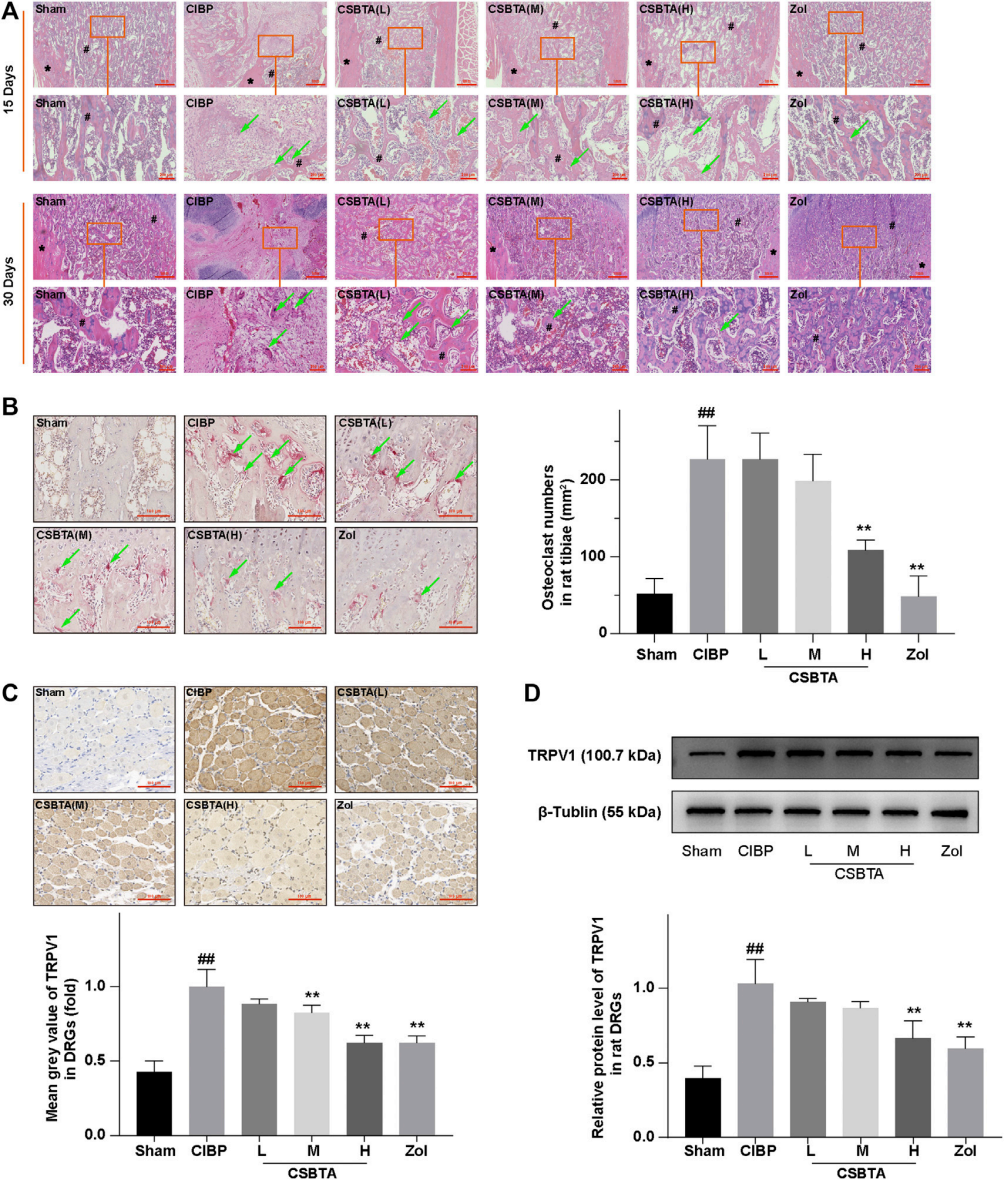
CSBTA ameliorated cancer cell-induced osteolysis and alleviated CIBP *in vivo*. **(A)** Decalcified sections from rat tibia were processed for HE staining at 15-days and 30-days after tumor cell inoculation. # marked the trabecular bone and * marked the cortical bone, the green arrow indicated osteoclast. Scale bar = 1 mm (low magnification) or 200 μm (high magnification). **(B)** Decalcified sections from rat tibiae were processed for TRAP staining at 30-days after tumor cell inoculation, and osteoclasts were counted and analyzed (*n* = 5). Scale bar = 100 µm. **(C)** Immunohistochemical analysis of the spread and expression of TRPV1 in DRGs of rats at 30-days after tumor cell inoculation (*n* = 3). Scale bar = 100 µm. **(D)** Western blot detection and densitometry quantification of TRPV1 expression in DRGs of rats at 30-days after tumor cell inoculation (*n* = 3). Data was presented as means ± SD. #*p* < 0.05, ##*p* < 0.01 vs. the Sham group. *p < 0.05, **p < 0.01 vs. the CIBP group.

Shenoy et al. (2017) reported that the histology could provide evidence for the cancer metastasis and osteolysis in the CIBP rat. Thus, here we investigated the protective effect of CSBTA by tibia HE and TRAP staining. HE staining of rat tibia sections showed that CIBP rat marrow cavity was invaded by cancer cells and trabecular bone also presented osteolysis on the 15th day (Figure 2A, circled in red). However, these above-mentioned situations got worse on the 30th day as cancer cells filled the marrow cavity and trabecular bone almost disappeared. In contrast, TRAP staining illustrated that osteolysis was slightly ameliorated in the CSBTA (L) and CSBTA (M) group and significantly suppressed in the CSBTA (H) group on the 30th day (Figure 2B). These results provided the *in vivo* evidence for the CSBTA-mediated inhibition of osteolysis.

Many molecules are related to allodynia, in turn, long term hyperalgesia often results in neurochemical changes in the primary afferent neurons. Previous studies reported that the elevation of TRPV1 in the DRGs was used as an indicator for nociceptor activation in response to CIBP (Yoneda et al., 2011; Maruyama et al., 2017). Consistent with the behavioral experiment, we also found a significant increase in the TRPV1 expression in DRGs at day 30 according to the result of immunohistochemical staining (Figure 2C). Compared to the CIBP group, medium-(50 mg/kg) and high-(100 mg/kg) dosage of CSBTA could obviously inhibit the TRPV1 overexpression in DRGs. Meanwhile, immunoblotting further confirmed that CSBTA could normalized the upregulation of TRPV1 in DRGs (Figure 2D). These results showed that CSBTA could ameliorate cancer cell-induced osteolysis and CIBP.

### Effect of CSBTA on Rat RANKL/OPG System

**FIGURE 3:**
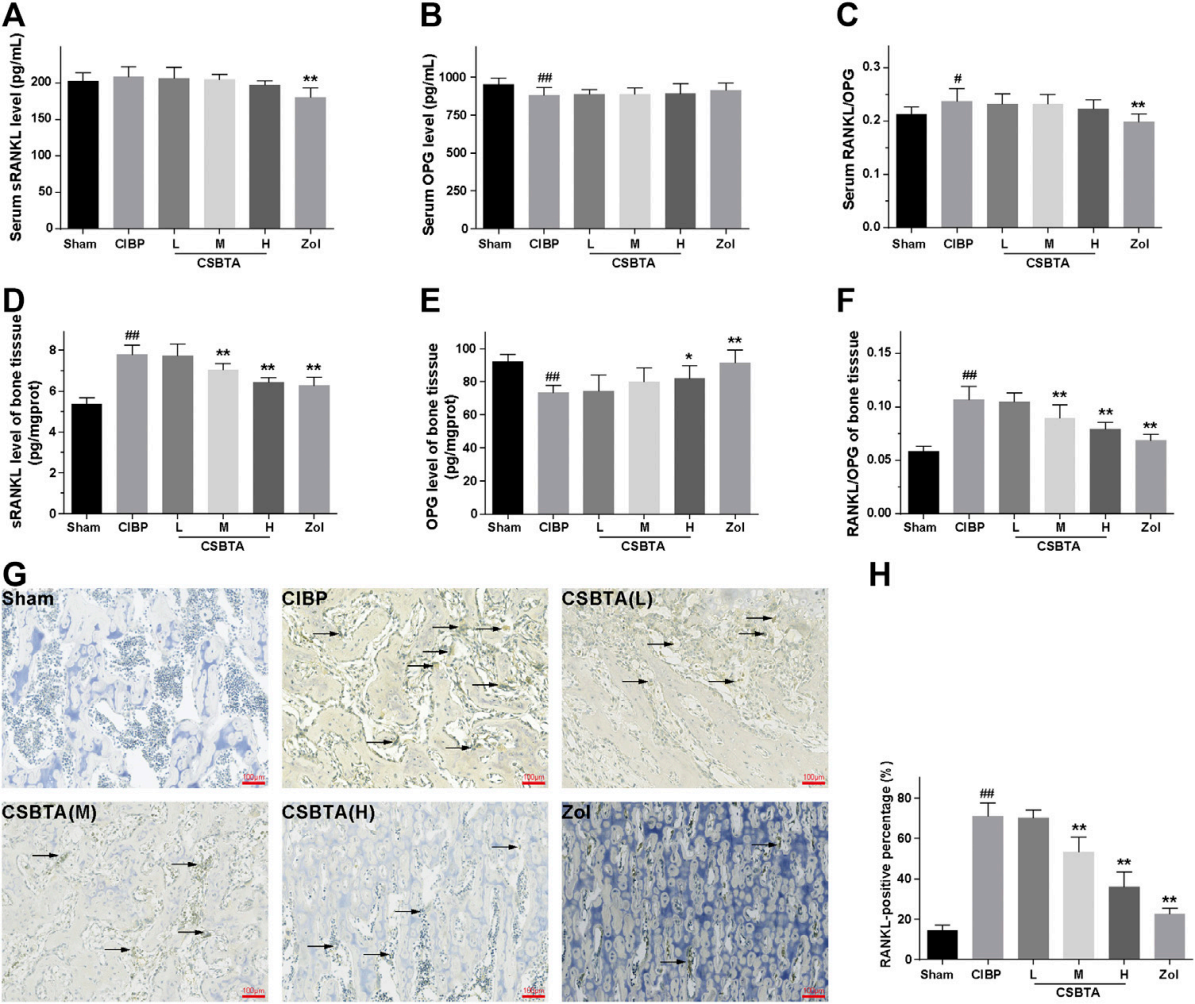
Effects of CSBTA on rat RANKL/OPG system at the 30th day after tumor cell inoculation. The level of soluble receptor activator of nuclear factor κB ligand (sRANKL, **(A)** and osteoprotegerin (OPG, **(B)** in the serum, OPG **(D)** and sRANKL **(E)** in the tibia tissue were measured by ELISA kit. sRANKL/OPG ratio in the serum **(C)** and tibiae **(F)** were further calculated (*n* = 6). **(G)** Immunohistochemical analysis of the spread of RANKL in tibiae of rats at 30-days after tumor cell inoculation (*n* = 5). **(H)**The RANKL-positive percentage were calculated (*n* = 5). Data was expressed as means ± SD and analyzed by one-way ANOVA. ^#^ *p* < 0.05, ^##^ *p* < 0.01 vs. the Sham group, *p < 0.05, **p < 0.01 vs. the CIBP group. Scale bar = 100 μm.

Ligand of receptor activator of nuclear factor kappa β (RANKL), a key stimulator of bone resorption, binds to receptor activator of nuclear factor kappa β (RANK) and situated on osteoclast precursors to aggravate osteoclastogenesis. OPG, a decoy receptor for RANKL, is a natural inhibitor of osteoclast formation (Park et al., 2018). As shown in Figure 3A, RANKL level was slightly elevated in the serum of CIBP rats without significant difference. After 30-days CSBTA treatment, there were no detectable changes in the serum RANKL compared to the CIBP group. Similarly, CSBTA treatment only caused mild up-regulation of OPG (Figure 3B). Besides, the serum RANKL/OPG was increased in CIBP rats (Figure 3C), while it was slightly decreased by CSBTA treatment. Interestingly, here we found that the RANKL/OPG balance of CIBP rat tibiae was impaired along with the dramatical increase of RANKL but significant decrease of OPG (Figures 3D–F). However, these impairments were reversed by the CSBTA treatment (Figures 3D–F). Correspondingly, immunohistochemical staining further confirmed that CSBTA exhibited strong capacity to down-regulate RANKL in tibia of CIBP rats as well (Figures 3G,H). These findings suggested that CSBTA was able to protect bone structure via normalizing the RANKL/OPG imbalance.

### Effects of CSBTA on Osteoclast Differentiation

**FIGURE 4:**
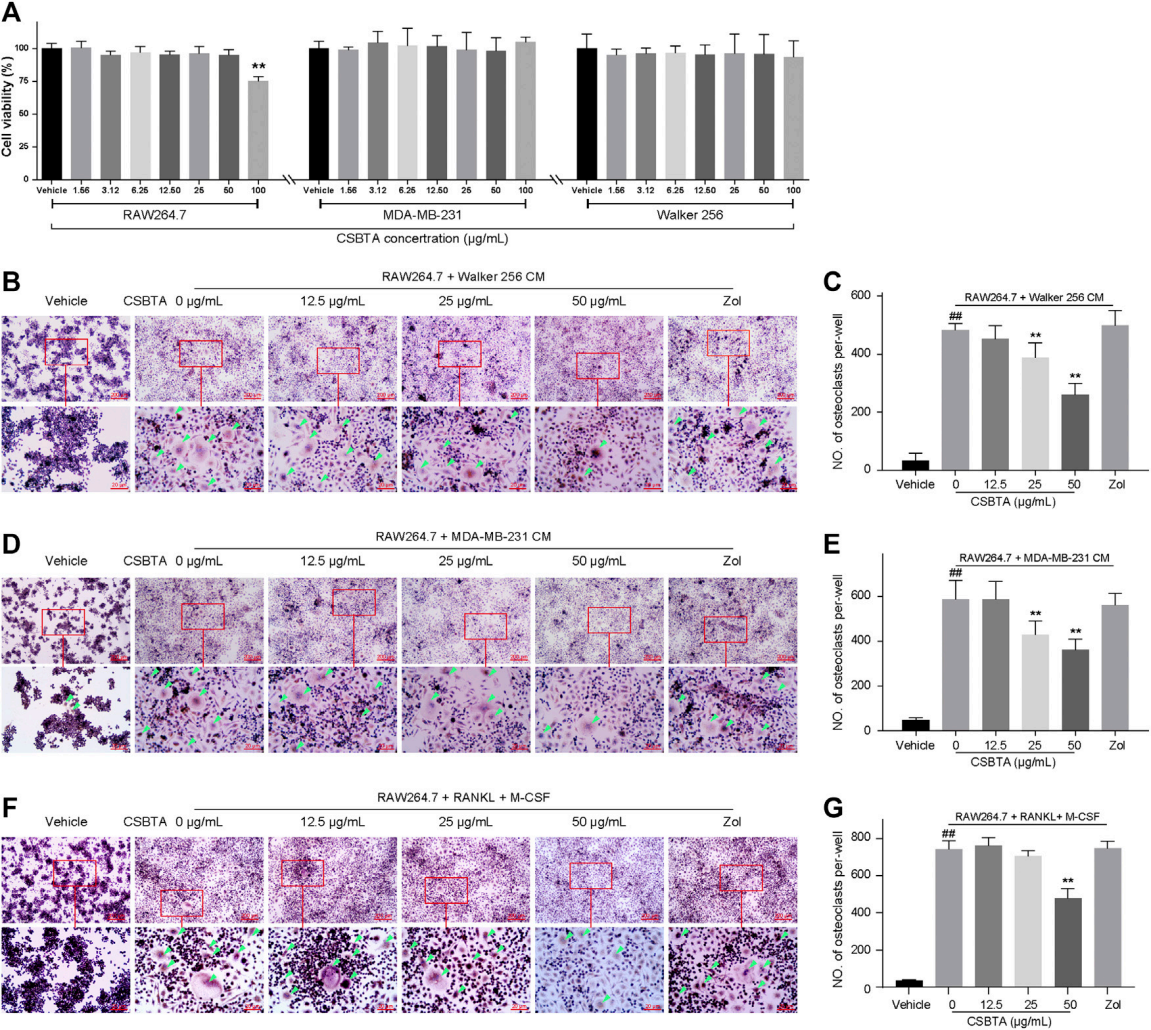
CSBTA inhibited cancer-induced osteoclastogenesis *in vitro*. **(A)** Viability of RAW 264.7, MDA-MB-231 and Walker 256 cells after incubation with CSBTA for 24 h (*n* = 6). **(B)** RAW 264.7 cells were stimulated by Walker 256 CM with or without varying concentrations of CSBTA for 4 days followed TRAP staining. **(C)** The number of TRAP positive cells of the Walker 256 CM induced RAW 264.7 cells per-well. **(D)** RAW 264.7 cells were stimulated by MDA-MB-231 CM with or without varying concentrations of CSBTA for 5 days followed TRAP staining. **(E)** The number of TRAP positive cells of the MDA-MB-231 CM induced RAW 264.7 cells per-well. **(F)** Effect of CSBTA treatment on osteoclast formation in RAW 264.7 (RAW) stimulated by sRANKL (100 ng/ml) and M-CSF (50 ng/ml) for 6 days. **(G)** The number of TRAP positive cells of the sRANKL (100 ng/ml) and M-CSF (50 ng/ml) induced RAW 264.7 cells per-well. The osteoclasts were marked by green triangles. All experiments were repeated at least three times. Data was expressed as means ± SD and analyzed by one-way ANOVA. ^#^ *p* < 0.05, ^##^ *p* < 0.01 *vs.* the Vehicle group. *p < 0.05, **p < 0.01 vs. the 0 μg/ml CSBTA group. Scale bar = 200 μm (low magnification) or 20 μm (high magnification).

Initially, MTT assay was performed to find the non-cytotoxic concentrations of CSBTA to RAW 264.7, MDA-MB-231 and Walker 256 cells. As shown in Figure 4A, up to 50 μg/ml, the concentrations of CSBTA were non-toxic to all the cell lines.

As skeletal metastasis is prevalent in most subtypes of breast cancer, the representative human breast cancer cell MDA-MB-231 was selected for the osteoclast differentiation assay. Osteoclast differentiation was assessed by TRAP staining after adding CM collected from MDA-MB-231 cells and Walker 256 cells to RAW 264.7 cells. As shown in Figures 4B–E, osteoclast differentiation was increased by CMs, while decreased by CSBTA treatment. Moreover, osteoclast differentiation stimulated by sRANKL + M-CSF cocktail was also inhibited by CSBTA (Figures 4F,G). These results indicated that CSBTA mediated the suppression of osteoclastogenesis *in vitro*.

### Effect of CSBTA on Cytokines in Breast Cancer Cells

**FIGURE 5:**
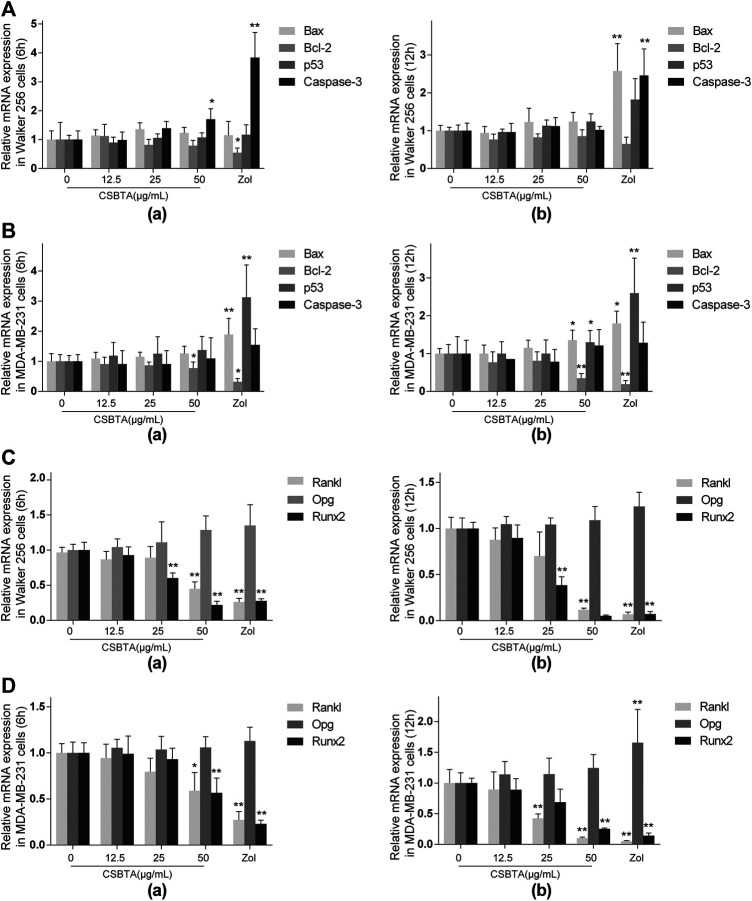
Effect of CSBTA on cytokines in breast cancer cells. The mRNA expression of apoptosis-associated genes Bax, Bcl-2, p53 and Caspase-3 of Walker 256 cells **(A)** and MDA-MB-231 **(B)** after treatment with CSBTA or Zoledronic acid (Zol) for 6 and 12 h. The mRNA expression of osteoclast-related genes Rankl, Opg and Runx2 of Walker 256 cells **(C)** and MDA-MB-231 cells **(D)** after treatment with CSBTA or Zol for 6 and 12 h. All experiments were repeated for 6 times. Data was expressed as means ± SD and analyzed by one-way ANOVA. *p < 0.05, **p < 0.01 vs. the 0 μg/ml CSBTA group.

There are many factors contributing to the bone metastasis, including invasion to the bone, recruitment of osteoclast precursors and the release of cytokine from the tumor cells (Le Pape et al., 2016; Pore et al., 2018). Previous studies have reported that CSBTA exhibited anti-cancer effects in many tumor cells (Zhang et al., 2016; Li et al., 2018). In order to justify whether CSBTA could inhibit breast cancer-induced osteoclast differentiation through its anticancer effects, here we compared the anticancer effects of CSBTA on the mRNA levels of Bax, Bcl-2, p53 and Caspase-3 in both breast cancer cell lines. As shown in (Figures 5A,B), CSBTA slightly increased the mRNA levels of Bax, p53, Caspase-3 and decreased Bcl-2 in both cell lines especially at the 50 μg/ml concentration**.** Runt-related transcription factor 2 (RUNX2), a transcription factor during embryogenesis, is aberrantly reactivated in many tumors and exacerbates bone resorption by upregulating the Rankl/Opg expression ratio (Sancisi et al., 2017; Yahiro et al., 2020). Here we found that CSBTA obviously decreased the mRNA levels of Rankl and Runx2 in both cell lines (Figures 5C,D **)**. Taken together, these findings highlighted that breast cancer-induced osteoclast differentiation was prevented by CSBTA through the suppression of Rankl in cancer cells.

### Effect of CSBTA on Expression and Secretion of sRANKL in Breast Cancer Cells

**FIGURE 6:**
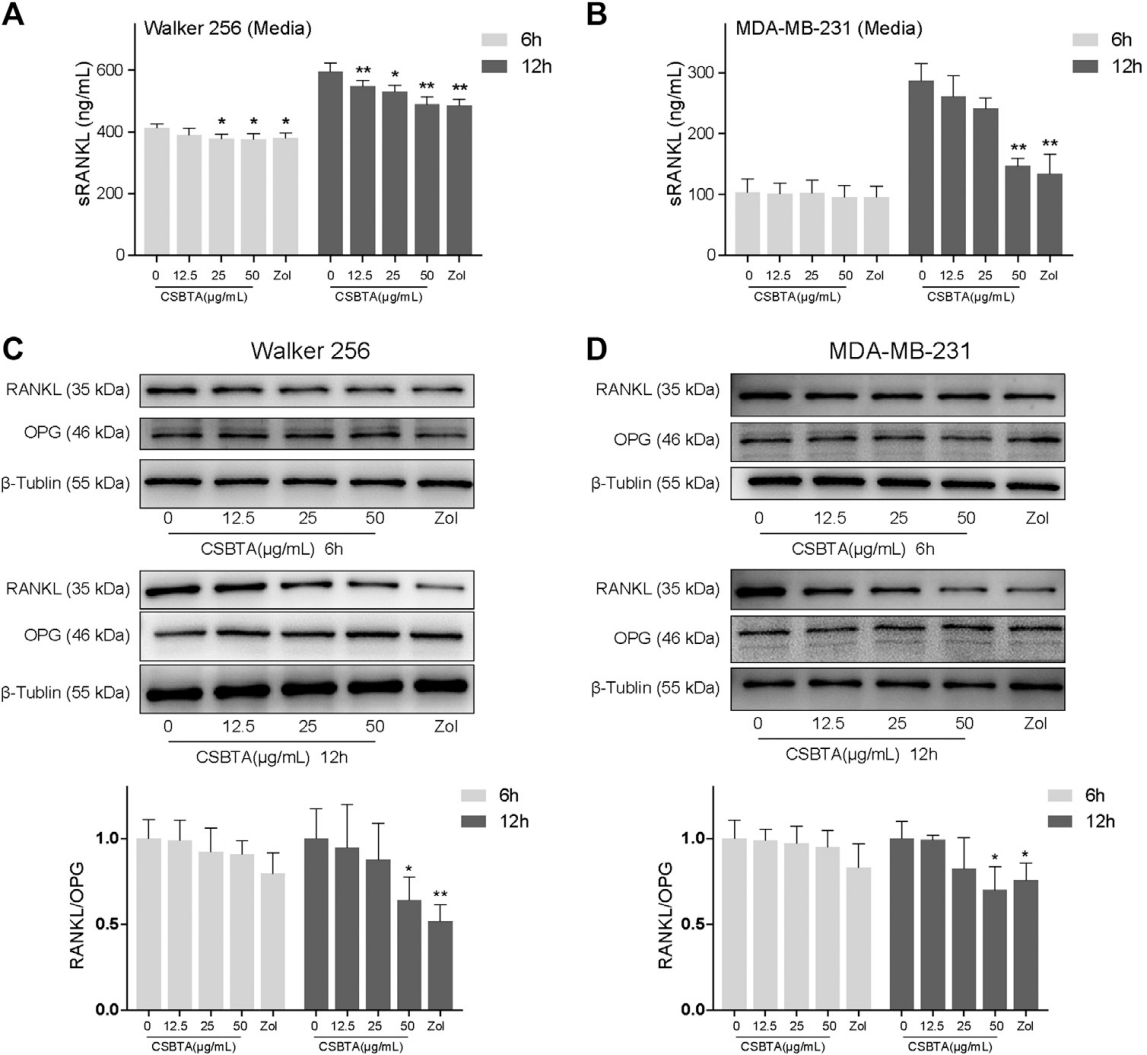
CSBTA treatment decreased sRANKL secretion and expression. Quantitation of sRANKL secretion of Walker 256 **(A)** and MDA-MB-231**(B)** cell media after treatment with CSBTA or Zol for 6 and 12 h. Immunoblotting for detection of RANKL, OPG and β-Tublin proteins in Walker 256 **(C)** and MDA-MB-231 **(D)** cells after treatment with CSBTA or Zol for 6 and 12 h. The histogram represented fold changes of relative proteins compared with 0 μg/ml CSBTA group. All experiments were repeated for 3 times. *p < 0.05, **p < 0.01 vs. the 0 μg/ml CSBTA group.

As presented in Figures 6A,B, exposure of Walker 256 and MDA-MB-231 cells to CSBTA led to a dramatically decrease in the secretion of sRANKL, particularly at 12 h. Immunoblotting also demonstrated that CSBTA inhibited RANKL protein levels in MDA-MB-231 and Walker 256 cell lines (Figures 6C,D). Although there was no significant change in the OPG level of both cells at all the time points, the RANKL/OPG ratio in each cell line still showed a significant decrease (Figures 6C,D). These results demonstrated that CSBTA inhibited the osteoclast differentiation through suppressing RANKL expression and secretion in breast cancer cells.

### CSBTA Abolished RANKL-Induced NF-κB Activation and c-Fos/NFATc1 Pathways in Osteoclastogenesis

**FIGURE 7:**
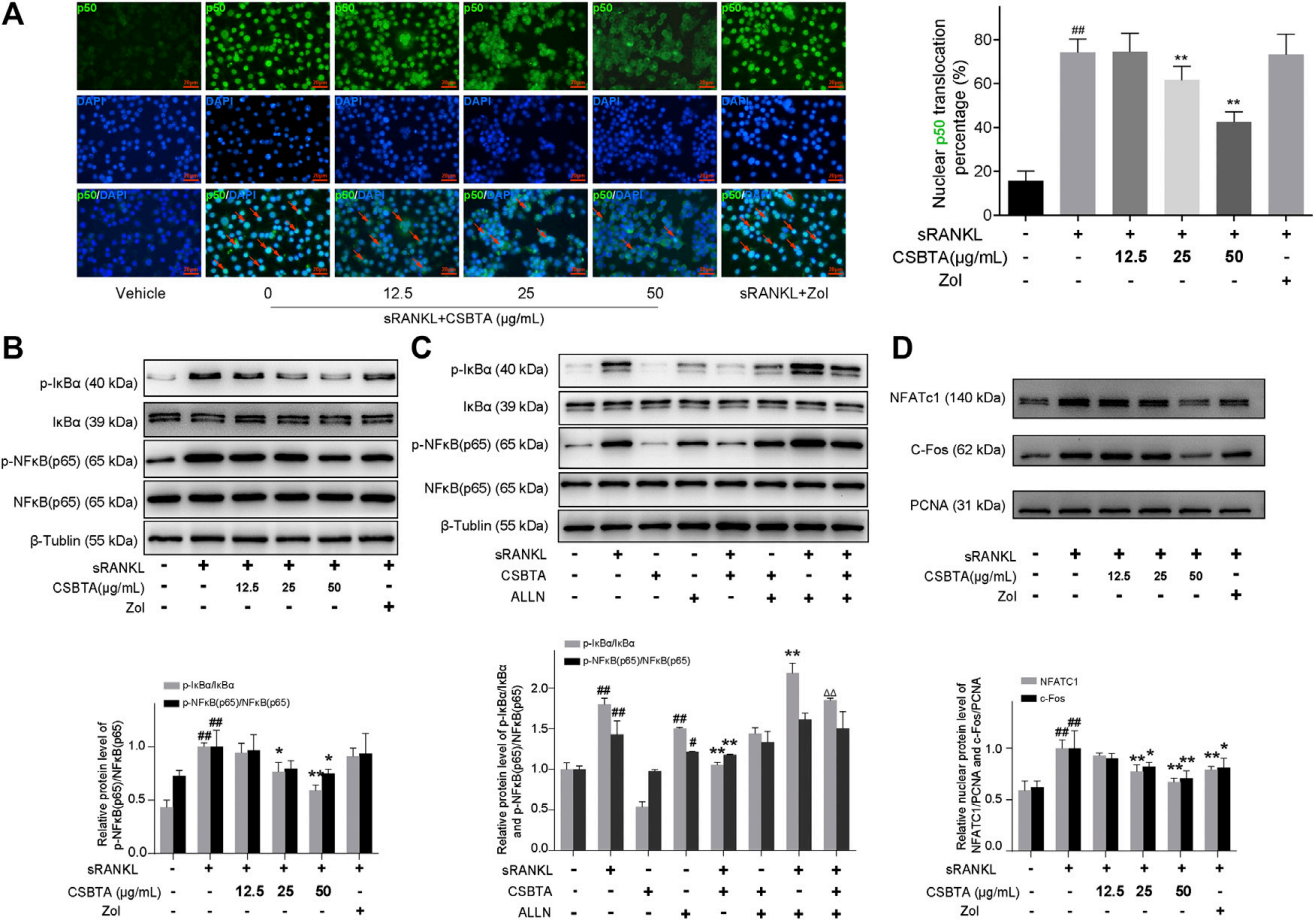
CSBTA suppressed RANKL-induced NF-κB activation and c-Fos/NFATc1 pathways in osteoclastogenesis. **(A)** RAW264.7 cells were double-stained for NF-κB (p50) in green, and DAPI in blue to detect the NF-κB (p50) localization after treatment with CSBTA. Scale bar = 20 μm. **(B)** RAW 264.7 cells were pretreated with CSBTA and followed by stimulation of sRANKL for 12 h to detect p-IκBα and p-NF-κB (p65) expression via Western-Blot. **(C)** RAW 264.7 cells were incubated with 50 μg/ml ALLN, CSBTA (50 μg/ml), sRANKL (10 nM) or the indicated combinations for 12 h to detect p-IκBα and p-NF-κB (p65) expression via Western-Blot. **(D)** RAW264.7 cells were pretreated with CSBTA and followed by stimulation with RANKL for 12 h to detect nuclear NFATc1, c-Fos expressions by Western Blot. The histogram represented fold changes of proteins. All experiments were repeated for 3 times. Data was expressed as means ± SD and analyzed by one-way ANOVA. ^#^ *p* < 0.05, ^##^ *p* < 0.01 sRANKL or ALLN vs. Vehicle, *p < 0.05, **p < 0.01 sRANKL/CSBTA or ALLN/CSBTA or sRANKL/ALLN *vs.* sRANKL, ^ΔΔ^ *p*< 0.01, sRANKL/CSBTA/ALLN *vs.* sRANKL/ALLN.

NF-κB, a key signal mediator of inflammatory and immune reactions, is also related to RANKL-induced osteoclast formation (Li et al., 2019). To investigate whether CSBTA could inhibit the activation of NF-κB by sRANKL, different concentrations of CSBTA-treated or untreated RAW 264.7 cells were exposed to sRANKL for 12 h. The immunofluorescence showed that NF-κB (p50) predominantly accumulated in the nucleus in RAW 264.7 cells after sRANKL stimulation (Figure 7A). However, after CSBTA treatment, NF-κB (p50) nuclear translocation was significantly inhibited compared to Vehicle cells. Normally, activation of NF-κB via RANKL requires the degradation of inhibitory subunit IκBα. Apparently, significant suppression of the p-NF-κB (p65) and p-IκBα induced by sRANKL was also observed at the 100 μg/ml concentration of CSBTA treatment (Figure 7B). Next, N-acetyl-leu-leu-norleucinal (ALLN), a kind of proteasome inhibitor, can inhibit the IκBα degradation. As shown in Figure 7C, sRANKL increased the phosphorylation levels of IκBα in RAW 264.7 cells, and the stimulation of ALLN further augmented the phosphorylation of IκBα (Park-Min, 2018). However, the levels of p-IκBa were reduced by CSBTA (50 μg/ml), and such phosphorylation could also not be observed when treated with CSBTA alone. Also, here we found the decrease of p-NF-κB (p65) induced by CSBTA was correlated to IκBα activation and such decrease could be inhibited by ALLN. Additionally, osteoclasts are special bone-resorbing cells which differentiate from monocyte/macrophage lineage cells in response to RANKL. During the osteoclastic maturation, transcription factors such as nuclear factor of activated T-cells c1 (NFATc1) and c-Fos function as a master regulator to activate osteoclastogenesis (Zhang et al., 2017). As shown in Figure 7D, nuclear protein levels of NFATc1 and c-Fos were evaluated by RANKL, while CSBTA significantly reduced the NFATc1 and c-Fos nuclear protein levels. Given above, CSBTA could suppress osteoclastogenesis by inhibiting RANKL-induced NF-κB and c-Fos/NFATc1 pathways.

**FIGURE 8:**
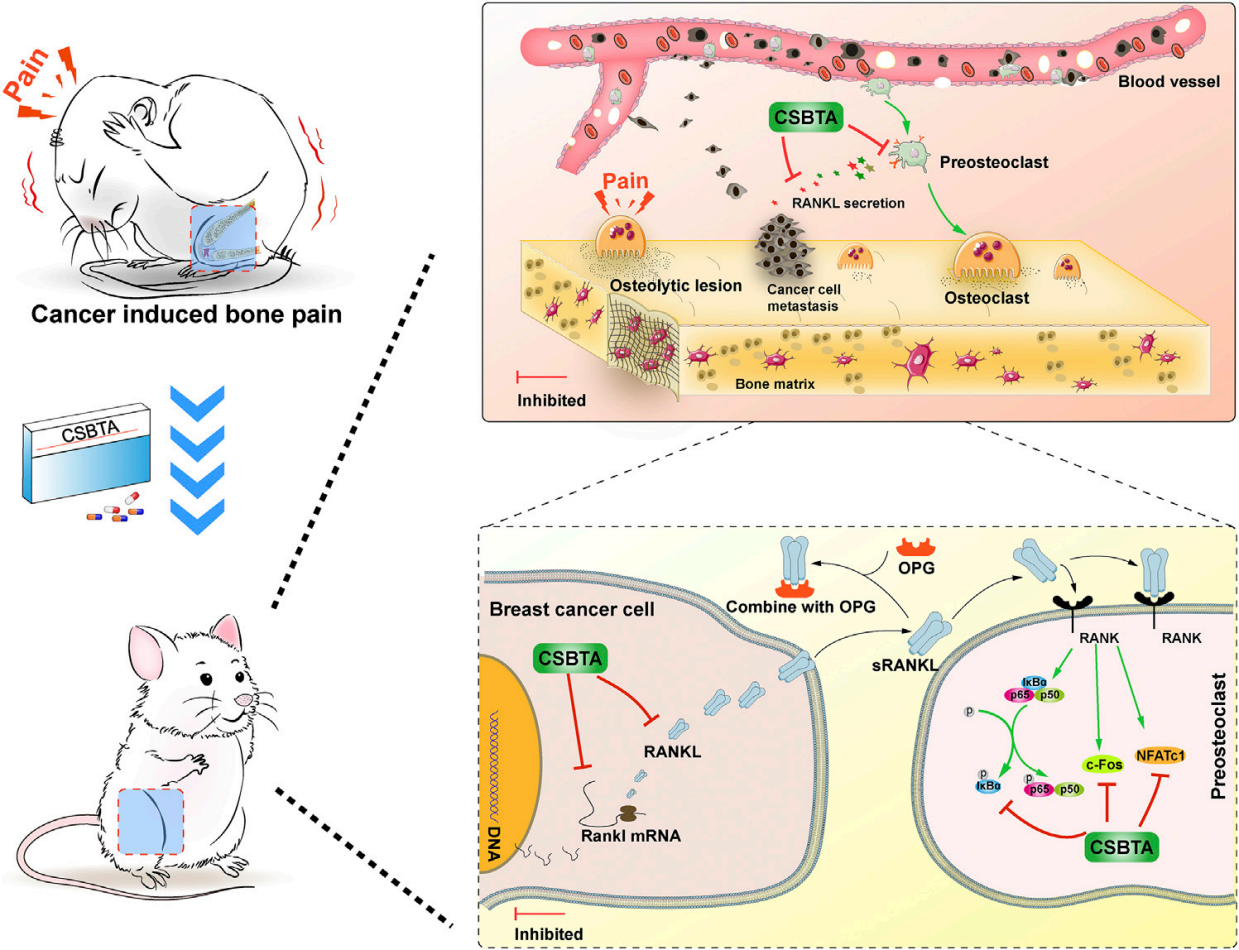
*Corydalis saxicola* bunting total alkaloids attenuates Walker 256-induced bone pain, osteoclastogenesis by suppressing RANKL-induced nuclear factor-κB and c-Fos/NFATc1 pathways in rats.

## Discussion

Natural products are the material basis for traditional Chinese medicine with the advantages of efficacy and safety (Rodrigues et al., 2016; Luo et al., 2019). It is suggested that natural compounds may provide alternative treatments for bone metastases related diseases owing to their inhibitory effects on tumor invasion and bone resorption (Doddapaneni et al., 2017; Wu et al., 2018; Yang et al., 2020b). *Corydalis saxicola Bunting total alkaloids*, extracted from the Chinese medicinal herb *C. saxicola* Bunting, was used for the treatment of hepatitis, bacterial infection, cancer, pain clinically in China (Wang et al., 2008; Liang et al., 2016; Liu et al., 2019). Our previous study showed that CSBTA could relieve cisplatin-induced neuropathic pain (Kuai et al., 2020). Breast cancer-induced bone pain is more complicated than other types of suffering, since it involves various factors, including the inflammatory factors, prostaglandins and disruption of bone homeostasis, *etc.* (Middlemiss et al., 2011). Osteolytic bone resorption is the main cause of pain (e.g., pathological bone fracture) in females with metastatic breast cancer (Pore et al., 2018). In addition, osteoclasts play a pivotal role in bone resorption dynamics (Zhang et al., 2019). Abnormal osteoclastic bone resorption is always associated with bone pain and antagonists of osteoclasts alleviate bone pain. (Nagae et al., 2006). Therefore, we suggest that the analgesic effect of CSBTA on the CIBP rat model may be related to the inhibition of the osteoclast.

Walker 256 induced bone cancer in rats is a typical animal model mimicking the physiological characteristics in patients with bone metastasis of breast cancer (Mao-Ying et al., 2006; Shenoy et al., 2017). Here the behavioral experimental results initially showed that CSBTA could improve CIBP in rats (Figure 1). And pathological results suggested that CSBTA could inhibit tumor-induced osteoclastogenesis in rats (Figure 2). Tissue damage and nerve injury often result in neurochemical changes in the primary afferent neurons. Apart from the behavioral experiment, previous researches have also underlined the relationship between CIBP and Transient receptor potential vanilloid subfamily 1 (TRPV1) (Li et al., 2014). TRPV1, a proton-activated cationic current-generating receptor, physiologically distributes in DRGs, periosteal as well as bone marrow nociceptive terminals (Niiyama et al., 2007; Scala et al., 2019). Therefore, overexpression of TRPV1 in DRGs is always an important indicator of CIBP (Fang et al., 2015). Consistent with the behavioral experiment, our data showed that the DRG TRPV1 was up-regulated in the CIBP rats while the overexpressed TRPV1 was downregulated by CSBTA (Figure 2). Meanwhile, immunohistochemistry and ELISA further confirmed that CSBTA significantly reduced RANKL secretion and osteoporosis in the tibial marrow cavity **(** Figure 3 **).** Taken together, these *in vivo* results suggested that CSBTA could ameliorate this CIBP and bone lesion by inhibiting breast cancer cell-induced osteoclast formation and function.

Efforts were then made to address the detailed mechanism by which CSBTA suppressed breast cancer cell-induced osteoclastogenesis. The relationship between RANKL/RANK and breast cancer has been reported by many publications (Sigl et al., 2016). Physiologically, RANKL binds to its natural receptor RANK, situated on osteoclasts or preosteoclast and initiated osteolysis, which mainly situates on osteoclasts or preosteoclast (Weichhaus et al., 2015; Li et al., 2019). However, metastatic breast cancer cells express excessive RANKL, which subsequently stimulate osteoclast differentiation from monocyte precursors. Thus, in the *in vitro* study, we employed murine macrophage cells (RAW 264.7) and conditioned medium from breast cancer cell (MDA-MB-231 and Walker 256 cells) to stimulate osteoclastic differentiation (Carina et al., 2018). CSBTA suppressed the osteoclastogenesis without affecting the viability of the RAW 264.7 cells up to 50 μg/ml, implicating that our study provided a direct method for the treatment of cancer cell metastasis related diseases **(** Figure 4 **)**. The subsequent *in vitro* results indicated that suppression of breast cancer-induced osteoclastogenesis with CSBTA was mainly accompanied by suppression of the mRNA levels of Rankl in cancer cells (Figure 5). Similar to the mRNA expression, here we found that CSBTA exhibited a significant inhibitory effect on the sRANKL/OPG of breast cancer cells (Figure 6).

It is well known that NF-κB activation is pro-inflammatory, characterized by induction of pro-inflammatory cytokines and result in hyperalgesia (Yang et al., 2020a). Besides, NF-κB, a critical transcription factor, is the early positive signal during RANKL-induced osteoclast differentiation (Maruyama et al., 2010; Kim et al., 2019). Under normal circumstance, the inhibitory subunit IκB binds to NF-κB, and subsequently prevents its nuclear translocation. Once IκB is phosphorylated and degraded, the activated-NF-κB is transported to the nucleus, binding with the DNA site and then initiates the transcription of the target genes. Being similar to the previous reports (Ahn et al., 2008; Wei et al., 2019), nuclear accumulation of NF-κB (p50) was observed after sRANKL stimulation in this study. In contrast, CSBTA remarkably prevented nuclear accumulation of NF-κB (p50) and inhibited the phosphorylation of NF-κB (p65), thus suppressed formation of osteoclast (Figures 7A,B). However, ALLN, a potent inhibitor of cysteine proteases, could reverse the decrease of p-IκBα or p-NF-κB (p65) with CSBTA (Figure 7C). Additionally, NFATc1 and c-Fos had been reported to be the paramount roles in RANKL-dependent osteoclastogenesis and subsequent osteolysis. NFATc1 activation facilitates the terminal differentiation of osteoclasts and c-Fos amplifies the expression of NFATc1 thus profoundly contributing to the process of osteoclastogenesis (Tran et al., 2019). Here we found that RANKL upregulated the nuclear c-Fos and NFATc1 after RANKL stimulation, which indicated that c-Fos and NFATc1 might also exert important effects on osteoclast formation. Intriguingly, CSBTA could decreased the nuclear protein levels of NFATc1 and c-Fos (Figure 7D). Therefore, CSBTA could inhibit osteoclastogenesis by suppressing RANKL-induced nuclear factor-κB and c-Fos/NFATc1 pathways.

In this study, we have demonstrated that (i) CSBTA relieved CIBP induced by Walker 256 cells; (ii) prevention of osteolytic bone resorption by CSBTA *in vivo* was accompanied by suppression of tibia RANKL level; (iii) CSBTA treatment decreased the mRNA levels of Rankl and osteoclast differentiation related cytokine in the mammary tumor cells; (iv) CSBTA decreased RANKL expression and secretion in breast cancer cells; (v) CSBTA could inhibit osteoclastogenesis by suppressing RANKL-induced nuclear factor-κB and c-Fos/NFATc1 pathways. To explore the molecular mechanisms involved in treatment of bone metastasis of breast cancer by CSBTA, future studies were required to elucidate the effects of CSBTA on ROS, ionic channel as well as autophagy in CIBP rats.

However, some limitations still exist in our study. Firstly, the effect of CSBTA on osteoblast, another kind of cell in bone tissue, was not clear enough. Next, this study did not distinguish the direct effect of CSBTA on the survival of tumor cells. Furthermore, this study did not include enough toxicological tests for animals. It definitely requires further investigation to deeply remedy these limitations. Overall, this study provides a promising therapeutic effect against cancer induced osteolysis and bone pain.

## Data Availability Statement

The original contributions presented in the study are included in the article/Supplementary Material, further inquiries can be directed to the corresponding authors.

## Ethics Statement

The animal study was reviewed and approved by Animal Ethics Committee of China Pharmaceutical University.

## Author Contributions

LJ: Viewing related literature, investigation, writing the original draft. PH: Data collation and statistics. PH, PC, JW, and ZL: Doing the experiment. FH and ZQ reviewed the manuscript. JC and FH: Funding acquisition, Supervision.

## Funding

This work was supported by the “National Science and Technology Major Project” from Ministry of Science and Technology of the People’s Republic of China (2017ZX09301026).

## Conflict of Interest

The authors declare that the research was conducted in the absence of any commercial or financial relationships that could be construed as a potential conflict of interest.

## Abbreviations

CSBTA, Corydalis saxicola Bunting total alkaloids; CIBP, cancer induced bone pain; RANKL, receptor activator of nuclear factor κ-B ligand; OPG, osteoprotegerin; M-CSF, macrophage colony stimulating factor; CM, conditioned media; ALLN, N-acetyl-leu-leu-norleucinal; DMSO, Dimethyl sulfoxide; MWT, mechanical withdrawal threshold; TRAP, tartrate-resistant acid phosphatase; TRPV1, transient receptor potential vanilloid subfamily, member 1; DRG, dorsal root ganglion; NF-κB, nuclear factor kappa-B; IκBα, inhibitor of NF-κB; NFATc1, nuclear factor of activated T-cells c1.

## References

[B1] Ahn K. S. Sethi G. Chaturvedi M. M. Aggarwal B. B. (2008). Simvastatin, 3-hydroxy-3-methylglutaryl coenzyme a reductase inhibitor, suppresses osteoclastogenesis induced by receptor activator of nuclear factor-kappaB ligand through modulation of NF-kappaB pathway. Int. J. Cancer. 123 (8), 1733–1740. 10.1002/ijc.23745 18688862

[B2] Aielli F. Ponzetti M. Rucci N. (2019). Bone metastasis pain, from the bench to the bedside. Int. J. Mol. Sci. 20 (2). 10.3390/ijms20020280 PMC6359191 30641973

[B3] An K. Rong H. Ni H. Zhu C. Xu L. Liu Q. (2018). Spinal PKC activation - induced neuronal HMGB1 translocation contributes to hyperalgesia in a bone cancer pain model in rats. Exp. Neurol. 303, 80–94. 10.1016/j.expneurol.2018.02.003 29428215

[B4] Bloom A. P. Jimenez-Andrade J. M. Taylor R. N. Castañeda-Corral G. Kaczmarska M. J. Freeman K. T. (2011). Breast cancer-induced bone remodeling, skeletal pain, and sprouting of sensory nerve fibers. J. Pain. 12 (6), 698–711. 10.1016/j.jpain.2010.12.016 21497141 PMC3111843

[B5] Carina V. Costa V. Pagani S. De Luca A. Raimondi L. Bellavia D. (2018). Inhibitory effects of low intensity pulsed ultrasound on osteoclastogenesis induced *in vitro* by breast cancer cells. J. Exp. Clin. Cancer Res. 37 (1), 197 10.1186/s13046-018-0868-2 30126457 PMC6102871

[B6] Cong Y. Sun K. He X. Li J. Dong Y. Zheng B. (2015). A traditional Chinese medicine Xiao-Ai-Tong suppresses pain through modulation of cytokines and prevents adverse reactions of morphine treatment in bone cancer pain patients. Mediat. Inflamm. 2015, 961635 10.1155/2015/961635 PMC4649101 26617438

[B7] Dai W. L. Yan B. Jiang N. Wu J. J. Liu X. F. Liu J. H. (2017). Simultaneous inhibition of NMDA and mGlu1/5 receptors by levo-corydalmine in rat spinal cord attenuates bone cancer pain. Int. J. Cancer. 141 (4), 805–815. 10.1002/ijc.30780 28500623

[B8] Dixon W. J. (1980). Efficient analysis of experimental observations. Annu. Rev. Pharmacol. Toxicol. 20, 441–462. 10.1146/annurev.pa.20.040180.002301 7387124

[B9] Doddapaneni R. Patel K. Chowdhury N. Singh M. (2017). Reversal of drug-resistance by noscapine chemo-sensitization in docetaxel resistant triple negative breast cancer. Sci. Rep. 7 (1), 15824 10.1038/s41598-017-15531-1 29158480 PMC5696458

[B10] Falk S. Appel C. K. Bennedbæk H. B. Al-Dihaissy T. Unger A. Dinkel K. (2019). Chronic high dose P2X7 receptor inhibition exacerbates cancer-induced bone pain. Eur. J. Pharmacol. 845, 48–55. 10.1016/j.ejphar.2018.12.032 30582910

[B11] Falk S. Dickenson A. H. (2014). Pain and nociception: mechanisms of cancer-induced bone pain. J. Clin. Oncol. 32 (16), 1647–1654. 10.1200/JCO.2013.51.7219 24799469

[B12] Fang D. Kong L. Y. Cai J. Li S. Liu X. D. Han J. S. (2015). Interleukin-6-mediated functional upregulation of TRPV1 receptors in dorsal root ganglion neurons through the activation of JAK/PI3K signaling pathway: roles in the development of bone cancer pain in a rat model. Pain. 156 (6), 1124–1144. 10.1097/j.pain.0000000000000158 25775359

[B13] Fornetti J. Welm A. L. Stewart S. A. (2018). Understanding the bone in cancer metastasis. J. Bone Miner. Res. 33 (12), 2099–2113. 10.1002/jbmr.3618 30476357

[B14] Fu P. Zhang Q. Yi D. Y. Abdelmaksoud A. Huang Q. Zhao H. Y. (2018). Dehydrocavidine attenuates d-galactose induced learning and memory impairment in rats. Neurosci. Lett. 665, 200–205. 10.1016/j.neulet.2017.12.004 29208407

[B15] Goldvaser H. Amir E. (2019). Role of bisphosphonates in breast cancer Therapy. Curr. Treat. Options Oncol. 20 (4, 26). 10.1007/s11864-019-0623-8 30874905

[B16] Hao Y. Gao R. Lu B. Ran Y. Yang Z. Liu J. (2018). Ghrelin protects against depleted uranium-induced bone damage by increasing osteoprotegerin/RANKL ratio. Toxicol. Appl. Pharmacol. 343, 62–70. 10.1016/j.taap.2018.02.015 29477364

[B17] Honore P. Luger N. M. Sabino M. A. Schwei M. J. Rogers S. D. Mach D. B. (2000). Osteoprotegerin blocks bone cancer-induced skeletal destruction, skeletal pain and pain-related neurochemical reorganization of the spinal cord. Nat. Med. 6 (5), 521–528. 10.1038/74999 10802707

[B18] Kim H. Kim B. Il Kim S. Kim H. J. Ryu B. Y. Chung J. (2019). S100A4 released from highly bone-metastatic breast cancer cells plays a critical role in osteolysis. Bone Res. 7, 30 10.1038/s41413-019-0068-5 31667000 PMC6804941

[B19] Kuai C. P. Ju L. J. Hu P. P. Huang F. (2020). *Corydalis saxicola* alkaloids attenuate cisplatin-induced neuropathic pain by reducing loss of IENF and blocking TRPV1 activation. Am. J. Chin. Med. 14, 1–22. 10.1142/S0192415X20500214 32138533

[B20] Lacey D. L. Boyle W. J. Simonet W. S. Kostenuik P. J. Dougall W. C. Sullivan J. K. (2012). Bench to bedside: elucidation of the OPG-RANK-RANKL pathway and the development of denosumab. Nat. Rev. Drug Discov. 11 (5), 401–419. 10.1038/nrd3705 22543469

[B21] Le Pape F. Vargas G. Clézardin P. (2016). The role of osteoclasts in breast cancer bone metastasis. J. Bone Oncol. 5 (3), 93–95. 10.1016/j.jbo.2016.02.008 27761364 PMC5063222

[B22] Lee B. H. Seong J. Kim U. J. Won R. Kim J. (2005). Behavioral characteristics of a mouse model of cancer pain. Yonsei Med. J. 46 (2), 252–259. 10.3349/ymj.2005.46.2.252 15861499 PMC2823022

[B23] Li M. Wang J. Mo B. Zeng J. Yao D. Chen F. (2018). Total alkaloids of *Corydalis saxicola* bunting inhibits migration of A549 cells by suppressing Cdc42 or Vav1. Oncol. Lett. 15 (1), 475–482. 10.3892/ol.2017.7273 29285198 PMC5738710

[B24] Li Y. Cai J. Han Y. Xiao X. Meng X. L. Su L. (2014). Enhanced function of TRPV1 via up-regulation by insulin-like growth factor-1 in a rat model of bone cancer pain. Eur. J. Pain. 18 (6), 774–784. 10.1002/j.1532-2149.2013.00420.x 24827675

[B25] Li Z. Zhu X. Xu R. Wang Y. Hu R. Xu W. (2019). Deacylcynaropicrin inhibits RANKL-induced osteoclastogenesis by inhibiting NF-κB and MAPK and promoting M2 polarization of macrophages. Front. Pharmacol. 10, 599 10.3389/fphar.2019.00599 31231214 PMC6567936

[B26] Liang Y. H. Tang C. L. Lu S. Y. Cheng B. Wu F. Chen Z. N. (2016). Serum metabonomics study of the hepatoprotective effect of *Corydalis saxicola* Bunting on carbon tetrachloride-induced acute hepatotoxicity in rats by (1)H NMR analysis. J. Pharmaceut. Biomed. Anal. 129, 70–79. 10.1016/j.jpba.2016.06.033 27399344

[B27] Liu X. Zheng H. Lu R. Huang H. Zhu H. Yin C. (2019). Intervening effects of total alkaloids of Corydalis saxicola bunting on rats with antibiotic-induced gut microbiota dysbiosis based on 16S rRNA gene sequencing and untargeted metabolomics analyses. Front. Microbiol. 10, 1151 10.3389/fmicb.2019.01151 31214133 PMC6555270

[B28] Luger N. M. Honore P. Sabino M. A. Schwei M. J. Rogers S. D. Mach D. B. (2001). Osteoprotegerin diminishes advanced bone cancer pain. Cancer Res. 61 (10), 4038–4047. 11358823

[B29] Luo H. Vong C. T. Chen H. Gao Y. Lyu P. Qiu L. (2019). Naturally occurring anti-cancer compounds: shining from Chinese herbal medicine. Chin. Med. 14, 48 10.1186/s13020-019-0270-9 31719837 PMC6836491

[B30] Luo J. Yang Z. Ma Y. Yue Z. Lin H. Qu G. (2016). LGR4 is a receptor for RANKL and negatively regulates osteoclast differentiation and bone resorption. Nat. Med. 22 (5), 539–546. 10.1038/nm.4076 27064449

[B31] Mao-Ying Q. L. Zhao J. Dong Z. Q. Wang J. Yu J. Yan M. F. (2006). A rat model of bone cancer pain induced by intra-tibia inoculation of Walker 256 mammary gland carcinoma cells. Biochem. Biophys. Res. Commun. 345 (4), 1292–1298. 10.1016/j.bbrc.2006.04.186 16725112

[B32] Maruyama K. Takayama Y. Kondo T. Ishibashi K. I. Sahoo B. R. Kanemaru H. (2017). Nociceptors boost the resolution of fungal osteoinflammation via the TRP channel-CGRP-Jdp2 Axis. Cell Rep. 19 (13), 2730–2742. 10.1016/j.celrep.2017.06.002 28658621

[B33] Maruyama T. Fukushima H. Nakao K. Shin M. Yasuda H. Weih F. (2010). Processing of the NF-kappa B2 precursor p100 to p52 is critical for RANKL-induced osteoclast differentiation. J. Bone Miner. Res. 25 (5), 1058–1067. 10.1359/jbmr.091032 19874202

[B34] Middlemiss T. Laird B. J. Fallon M. T. (2011). Mechanisms of cancer-induced bone pain. Clin. Oncol. 23 (6), 387–392. 10.1016/j.clon.2011.03.003 21683564

[B35] Nagae M. Hiraga T. Wakabayashi H. Wang L. Iwata K. Yoneda T. (2006). Osteoclasts play a part in pain due to the inflammation adjacent to bone. Bone. 39 (5), 1107–1115. 10.1016/j.bone.2006.04.033 16769263

[B36] Nagae M. Hiraga T. Yoneda T. (2007). Acidic microenvironment created by osteoclasts causes bone pain associated with tumor colonization. J. Bone Miner. Metabol. 25 (2), 99–104. 10.1007/s00774-006-0734-8 17323179

[B37] Niiyama Y. Kawamata T. Yamamoto J. Omote K. Namiki A. (2007). Bone cancer increases transient receptor potential vanilloid subfamily 1 expression within distinct subpopulations of dorsal root ganglion neurons. Neuroscience. 148 (2), 560–572. 10.1016/j.neuroscience.2007.05.049 17656027

[B38] Park S. H. Eber M. R. Widner D. B. Shiozawa Y. (2018). Role of the bone microenvironment in the development of painful complications of skeletal metastases. Cancers (Basel). 10 (5). 10.3390/cancers10050141 PMC5977114 29747461

[B39] Park-Min K. H. (2018). Mechanisms involved in normal and pathological osteoclastogenesis. Cell. Mol. Life Sci. 75 (14), 2519–2528. 10.1007/s00018-018-2817-9 29670999 PMC9809143

[B40] Pore S. K. Hahm E. R. Latoche J. D. Anderson C. J. Shuai Y. Singh S. V. (2018). Prevention of breast cancer-induced osteolytic bone resorption by benzyl isothiocyanate. Carcinogenesis. 39 (2), 134–145. 10.1093/carcin/bgx114 29040431 PMC5862255

[B41] Rodrigues T. Reker D. Schneider P. Schneider G. (2016). Counting on natural products for drug design. Nat. Chem. 8 (6), 531–541. 10.1038/nchem.2479 27219696

[B42] Sancisi V. Manzotti G. Gugnoni M. Rossi T. Gandolfi G. Gobbi G. (2017). RUNX2 expression in thyroid and breast cancer requires the cooperation of three non-redundant enhancers under the control of BRD4 and c-JUN. Nucleic Acids Res. 45 (19), 11249–11267. 10.1093/nar/gkx802 28981843 PMC5737559

[B43] Scala R. Maqoud F. Angelelli M. Latorre R. Perrone M. G. Scilimati A. (2019). Zoledronic acid modulation of TRPV1 channel currents in osteoblast cell line and native rat and mouse bone marrow-derived osteoblasts: cell proliferation and mineralization effect. Cancers (Basel). 11 (2). 10.3390/cancers11020206 PMC6406412 30754651

[B44] Shenoy P. Kuo A. Vetter I. Smith M. T. (2017). Optimization and in vivo profiling of a refined rat model of walker 256 breast cancer cell-induced bone pain using behavioral, radiological, histological, immunohistochemical and pharmacological methods. Front. Pharmacol. 8, 442 10.3389/fphar.2017.00442 28729837 PMC5498471

[B45] Sigl V. Jones L. P. Penninger J. M. (2016). RANKL/RANK: from bone loss to the prevention of breast cancer. Open Biol. 6 (11). 10.1098/rsob.160230 PMC5133443 27881737

[B46] Tran P. T. Dat N. T. Dang N. H. Van Cuong P. Lee S. Hwangbo C. (2019). Ganomycin I from Ganoderma lucidum attenuates RANKL-mediated osteoclastogenesis by inhibiting MAPKs and NFATc1. Phytomedicine. 55, 1–8. 10.1016/j.phymed.2018.10.029 30668419

[B47] Wang T. Sun N. L. Zhang W. D. Li H. L. Lu G. C. Yuan B. J. (2008). Protective effects of dehydrocavidine on carbon tetrachloride-induced acute hepatotoxicity in rats. J. Ethnopharmacol. 117 (2), 300–308. 10.1016/j.jep.2008.02.010 18358653

[B48] Wei G. Liang T. Wei C. Nong X. Lu Q. Zhao J. (2019). Daidzin inhibits RANKL-induced osteoclastogenesis *in vitro* and prevents LPS-induced bone loss *in vivo* . J. Cell. Biochem. 120 (4), 5304–5314. 10.1002/jcb.27806 30378146

[B49] Weichhaus M. Chung S. T. Connelly L. (2015). Osteoprotegerin in breast cancer: beyond bone remodeling. Mol. Canc. 14, 117 10.1186/s12943-015-0390-5 PMC4460694 26054853

[B50] Wu F. Zheng H. Yang Z. T. Cheng B. Wu J. X. Liu X. W. (2017). Urinary metabonomics study of the hepatoprotective effects of total alkaloids from *Corydalis saxicola* Bunting on carbon tetrachloride-induced chronic hepatotoxicity in rats using. J. Pharmaceut. Biomed. Anal. 140, 199–209. 10.1016/j.jpba.2017.03.031 28363136

[B51] Wu Q. Zheng K. Huang X. Li L. Mei W. (2018). Tanshinone-IIA-based analogues of imidazole alkaloid act as potent inhibitors to block breast cancer invasion and metastasis *in vivo* . J. Med. Chem. 61 (23), 10488–10501. 10.1021/acs.jmedchem.8b01018 30398868

[B52] Yahiro Y. Maeda S. Morikawa M. Koinuma D. Jokoji G. Ijuin T. (2020). BMP-induced Atoh8 attenuates osteoclastogenesis by suppressing Runx2 transcriptional activity and reducing the Rankl/Opg expression ratio in osteoblasts. Bone Res. 8, 32 10.1038/s41413-020-00106-0 32923015 PMC7463266

[B53] Yang B. Zhang Z. Yang Z. Ruan J. Luo L. Long F. (2020a). Chanling Gao attenuates bone cancer pain in rats by the IKKβ/NF-κB signaling pathway. Front. Pharmacol. 11, 525 10.3389/fphar.2020.00525 32431607 PMC7214814

[B54] Yang M. Xie J. Lei X. Song Z. Gong Y. Liu H. (2020b). Tubeimoside I suppresses diabetes-induced bone loss in rats, osteoclast formation, and RANKL-induced nuclear factor-kappaB pathway. Int. Immunopharm. 80, 106202 10.1016/j.intimp.2020.106202 32004923

[B55] Yoneda T. Hata K. Nakanishi M. Nagae M. Nagayama T. Wakabayashi H. (2011). Involvement of acidic microenvironment in the pathophysiology of cancer-associated bone pain. Bone. 48 (1), 100–105. 10.1016/j.bone.2010.07.009 20637323

[B56] Yu J. Liu Q. Lu X. Li X. Li N. Liu B. (2018). Inhibitory and inductive effects of *Corydalis saxicola* Bunting total alkaloids (CSBTA) on cytochrome P450s in rats. Phytother Res. 7, 33–39. 10.1002/ptr.6117 29806105

[B57] Zeng F. L. Xiang Y. F. Liang Z. R. Wang X. Huang D. E. Zhu S. N. (2013). Anti-hepatitis B virus effects of dehydrocheilanthifoline from *Corydalis saxicola* . Am. J. Chin. Med. 41 (1), 119–130. 10.1142/S0192415X13500092 23336511

[B58] Zhang B. Huang R. Hua J. Liang H. Pan Y. Dai L. (2016). Antitumor lignanamides from the aerial parts of *Corydalis saxicola* . Phytomedicine. 23 (13), 1599–1609. 10.1016/j.phymed.2016.09.006 27823624

[B59] Zhang Y. Xu S. Li K. Tan K. Liang K. Wang J. (2017). mTORC1 inhibits NF-κB/NFATc1 signaling and prevents osteoclast precursor differentiation, in vitro and in mice. J. Bone Miner. Res. 32 (9), 1829–1840. 10.1002/jbmr.3172 28520214

[B60] Zhang Y. Zou B. Tan Y. Su J. Wang Y. Xu J. (2019). Sinomenine inhibits osteolysis in breast cancer by reducing IL-8/CXCR1 and c-Fos/NFATc1 signaling. Pharmacol. Res. 142, 140–150. 10.1016/j.phrs.2019.02.015 30797069

