# Supplementary material for: Corydalis Saxicola Bunting Total Alkaloids Attenuate Walker 256-Induced Bone Pain and Osteoclastogenesis by Suppressing RANKL-Induced NF-κB and c-Fos/NFATc1 Pathways in Rats
Source: Front Pharmacol. 2021 Jan 26;11:609119. doi: 10.3389/fphar.2020.609119 (PMC7870471; doi:10.3389/fphar.2020.609119)
Supplement: Supplementary file 1 [file datasheet1.docx]

***Supplementary Material***

**1 Supplementary tables：**

**Table S1. Limb use score of the rat**

| **Points** | **Main features** |
| --- | --- |
| **0** | Total lack of limb use |
| **1** | Significant limping and partial lack of limb use |
| **2** | Significant limping |
| **3** | Insignificant limping |
| **4** | Normal use of hind limb |

**Table S2. Information of PCR primers**

| **Walker 256 cells** | | **Primer sequences** |
| --- | --- | --- |
| **Bax** | **Forward** | 5' CCAGGACGCATCCACCAAGAAG 3' |
|  | **Reverse** | 5' GCTGCCACACGGAAGAAGACC 3' |
| **Bcl-2** | **Forward** | 5' GCCTGAGAGCAACCGAACGC 3' |
|  | **Reverse** | 5' AGGTGGCACAGGGCTGAGC3' |
| **p53** | **Forward** | 5' TGGGACGGGACAGCTTTGAGG 3' |
|  | **Reverse** | 5' CTGGTGGGCAGTGCTCTCTTTG 3' |
| **Caspase-3** | **Forward** | 5' TTTGGAACGAACGGACCTGTGG 3' |
|  | **Reverse** | 5' ACCGCAGTCCAGCTCTGTACC 3' |
| **Runx2** | **Forward** | 5' CCGCACGACAACCGCACCAT 3' |
|  | **Reverse** | 5' CGCTCCGGCCCACAAATCTC 3' |
| **Rankl** | **Forward** | 5' ACGCAGATTTGCAGGACTCGAC3' |
|  | **Reverse** | 5' TTCGTGCTCCCTCCTTTCATC 3' |
| **Opg** | **Forward** | 5' TGGCACACGAGTGATGAATGCG 3' |
|  | **Reverse** | 5' GCTGGAAAGTTTGCTCTTGCG 3' |
| **Gapdh** | **Forward** | 5' GATGCTGGTGCTGAGTATGRCG 3' |
|  | **Reverse** | 5' GTGGTGCAGGATGCATTGCTCTGA 3' |
| **MDA-MB-231 cells** | | **Primer sequences** |
| **Bax** | **Forward** | 5' CGAACTGGACAGTAACATGGAG3' |
|  | **Reverse** | 5' CAGTTTGCTGGCAAAGTAGAAA3' |
| **Bcl-2** | **Forward** | 5' GACTTCGCCGAGATGTCCAG 3' |
|  | **Reverse** | 5' GAACTCAAAGAAGGCCACAATC3' |
| **p53** | **Forward** | 5' TTCCTGAAAACAACGTTCTGTC3' |
|  | **Reverse** | 5'AACCATTGTTCAATATCGTCCG3' |
| **Caspase-3** | **Forward** | 5'CCAAAGATCATACATGGAAGCG3' |
|  | **Reverse** | 5'CTGAATGTTTCCCTGAGGTTTG3' |
| **Runx2** | **Forward** | 5'TCAACGATCTGAGATTTGTGGG3' |
|  | **Reverse** | 5'GGGGAGGATTTGTGAAGACGG3' |
| **Rankl** | **Forward** | 5'TTACCTGTATGCCAACATTTGC3' |
|  | **Reverse** | 5'TTTGATGCTGGTTTTAGTGACG3' |
| **Opg** | **Forward** | 5'GAAACGTTTCCTCCAAAGTACC3' |
|  | **Reverse** | 5'CTGTCTGTGTAGTAGTGGTCAG3' |
| **Gapdh** | **Forward** | 5'CAGGAGGCATTGCTGATGAT3' |
|  | **Reverse** | 5'GAAGGCTGGGGCTCATTT3' |

**2 Supplementary figures：**

**
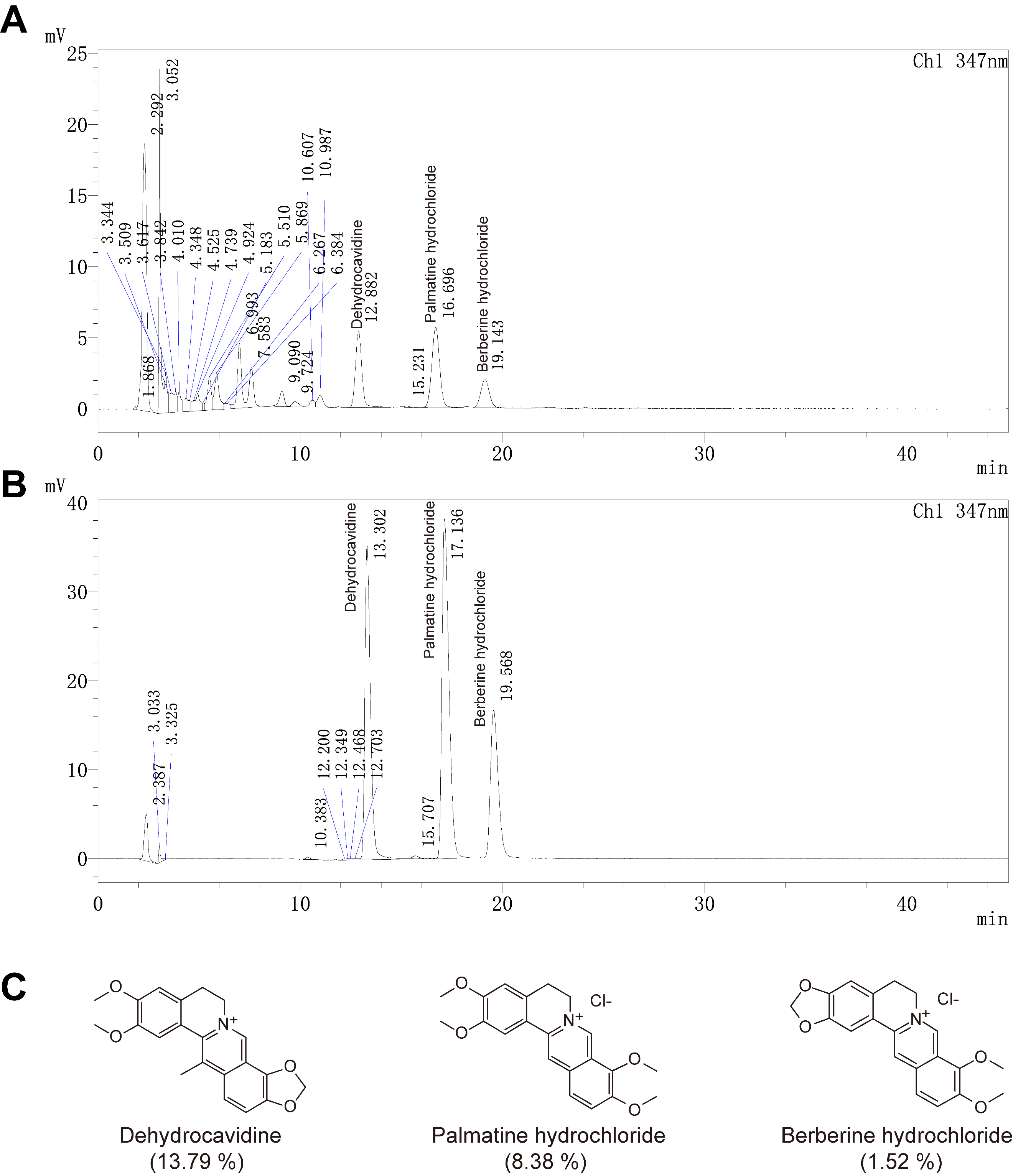
**

**Figure S1. HPLC chromatograms of CSBTA.** **(A)** HPLC chromatograms of test solution detected at 347 nm. **(B)** HPLC chromatograms of reference solution detected at 347 nm. **(C)** The structure and content of dehydrocarbamate, palmatine hydrochloride and berberine hydrochloride in CSBTA.

**
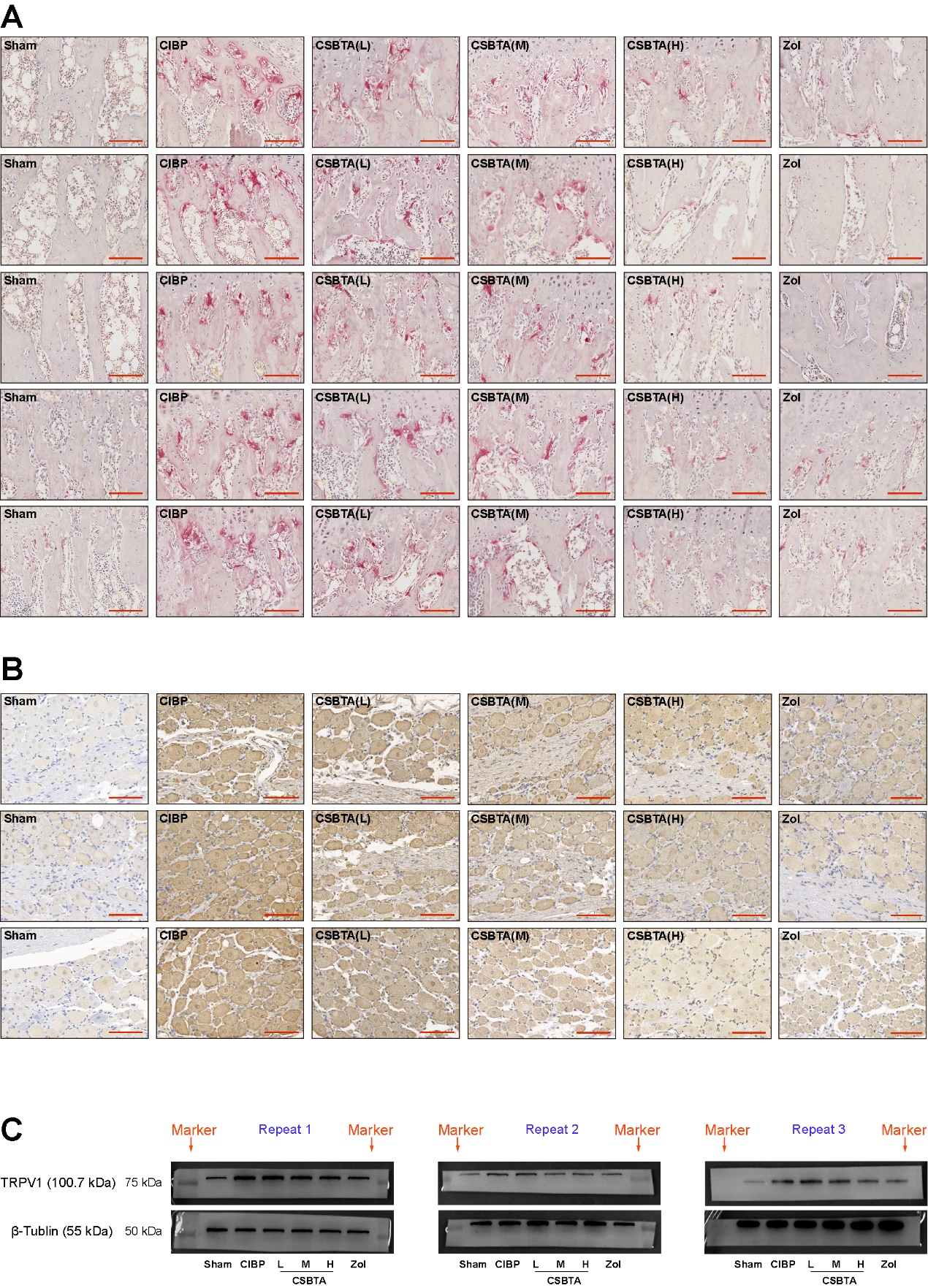
**

**Figure S2.** **(A)** Original TRAP staining images from rat tibia sections. (Images of Figure 2B in the article, scale bar=100 µm)**.** **(B)** Original TRPV1 immunohistochemical staining images from rat dorsal root ganglion sections. (Images of **Figure 2C** in the article, scale bar=100 µm). **(C)** Original western blot for three repeats to show the effect of CSBTA on rat DRG TRPV1 expression. (Images of **Figure 2D** in the article)

**
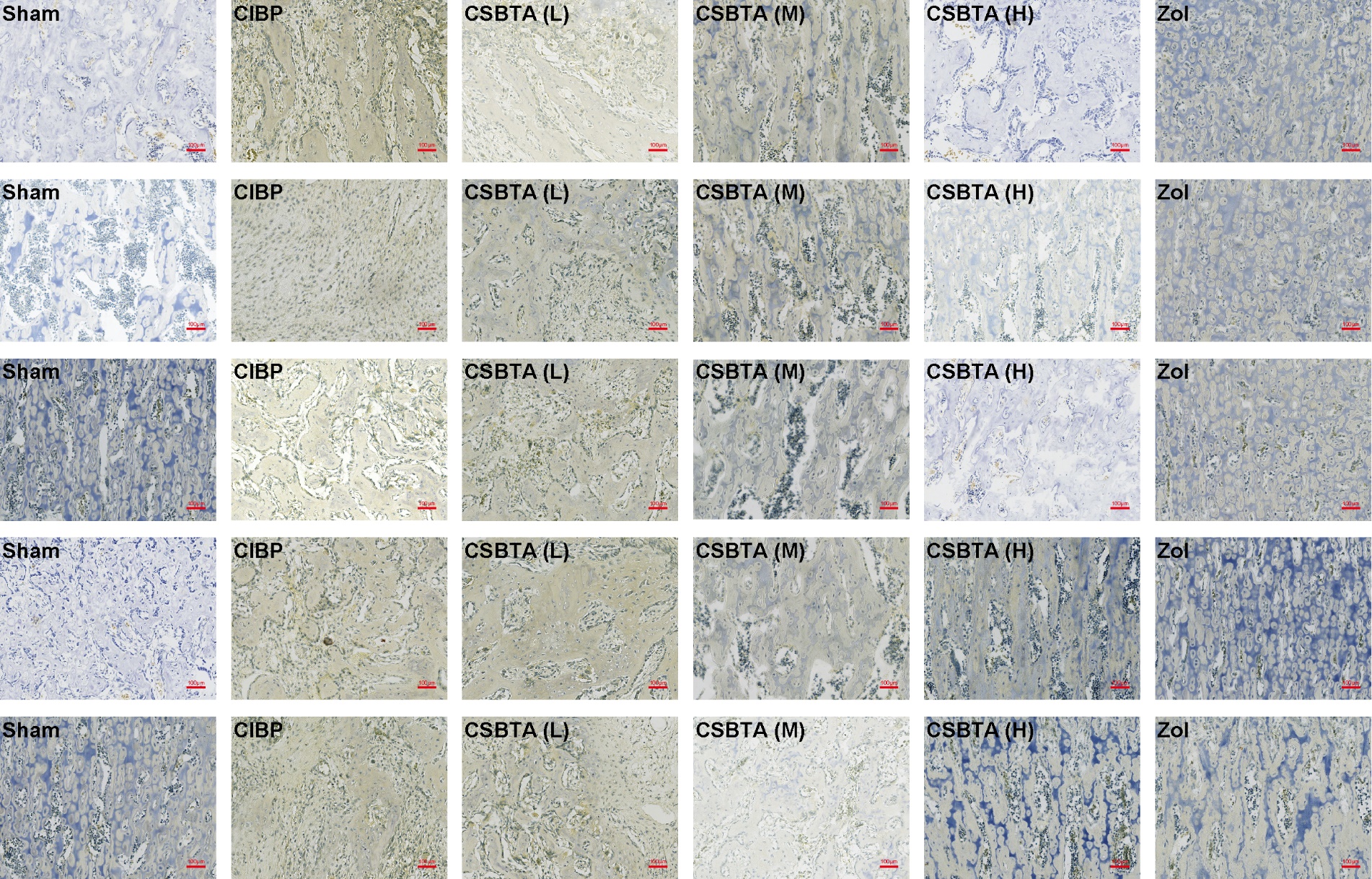
**

**Figure S3.** Original RANKL immunohistochemical staining images from rat tibia sections**.** (Images of **Figure 3G** in the article)**.**


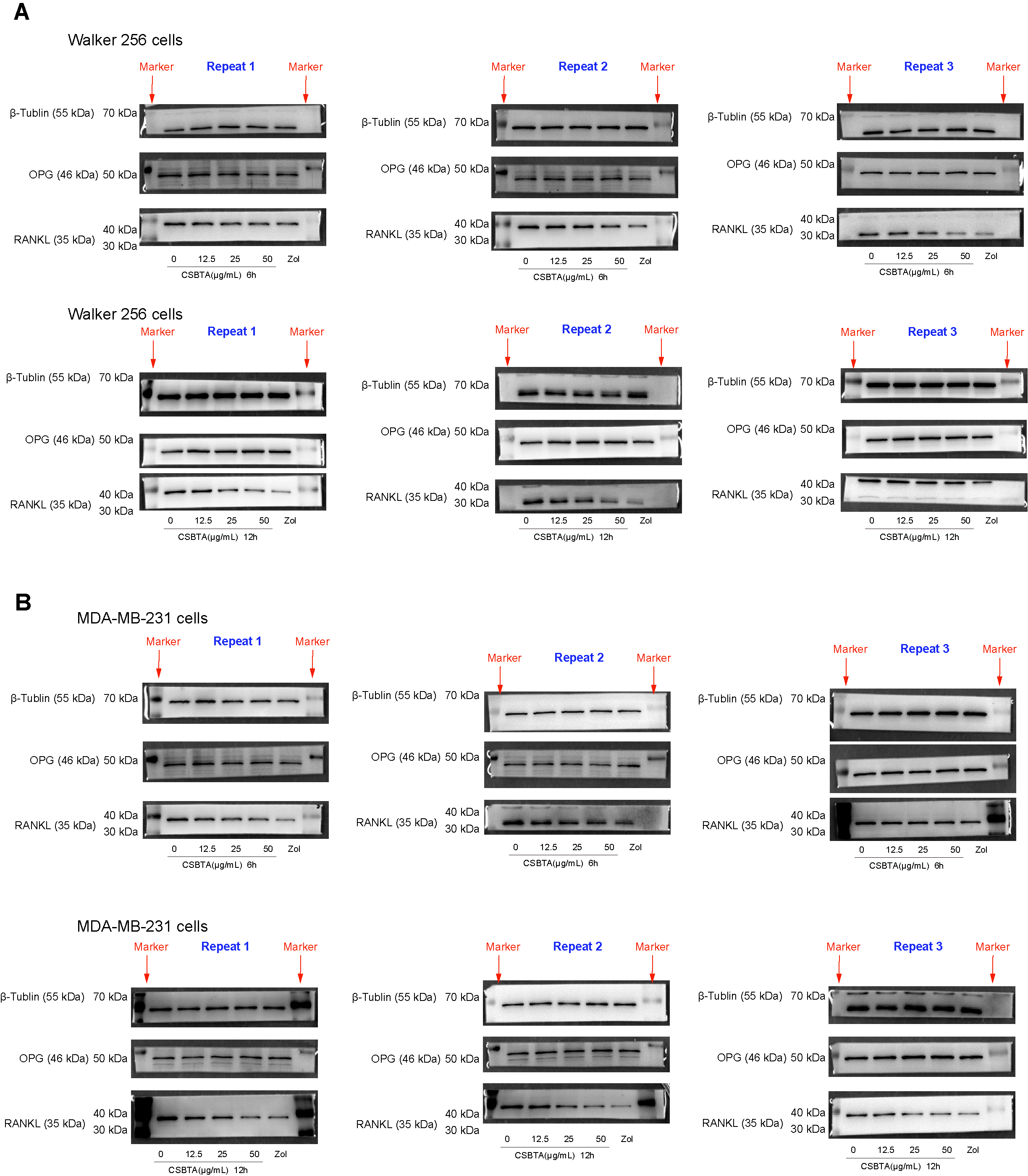


**Figure S4.** (A) Original western blot for three repeats to show the effect of CSBTA on the RANKL/OPG system in Walker 256 cells. (Western Blot images of **Figure 6 C** in the article) (B) Original western blot for three repeats to show the effect of CSBTA on the RANKL/OPG system in MDA-MB-231 cells. (Western Blot images of **Figure 6 D** in the article). The whole blot membranes were cut at molecular weight of 70 kDa, 50 kDa and 30~40 kDa for β-Tublin (55 kDa), OPG (46 kDa), RANKL (35 kDa).

**
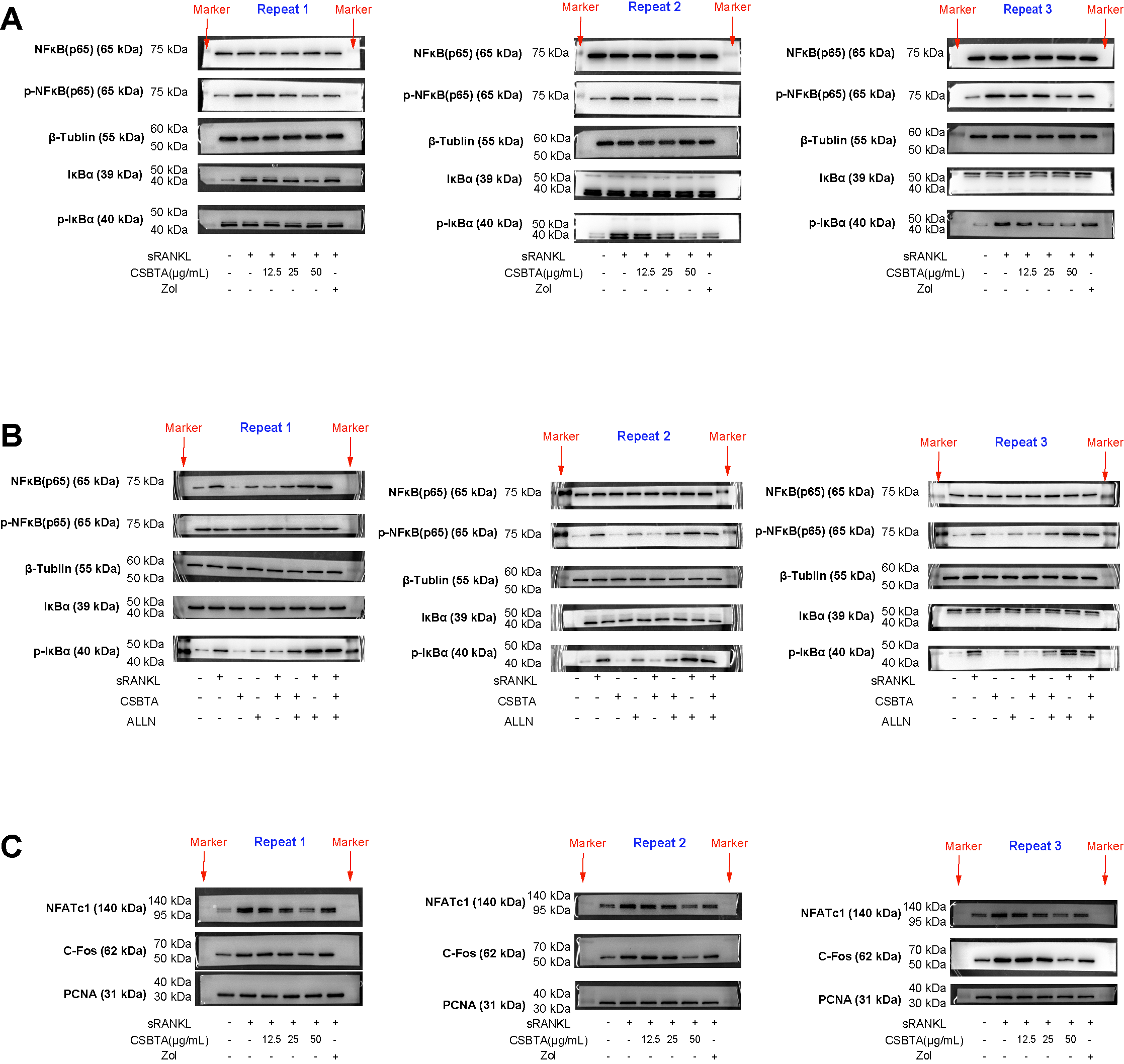
**

**Figure S5. (A)** Original western blot for three repeats to show the effects of CSBTA on the RANKL induced IκBα and NFκB (p65) activation in RAW264.7 cells. (Western Blot images of **Figure 7B** in the article). **(B)** Original western blot for three repeats to show CSBTA abolished RANKL-induced NF-κB activation via inhibiting IκBα phosphorylation (Western Blot images of **Figure 7C** in the article). **(D)** Original western blot for three repeats to show the effects of CSBTA on nuclear NFATc1, c-Fos expressions in RAW264.7 cells (Western Blot images of **Figure 7D** in the article).
